# Generation of LexA enhancer-trap lines in *Drosophila* by an international scholastic network

**DOI:** 10.1093/g3journal/jkad124

**Published:** 2023-06-06

**Authors:** Ella S Kim, Arjun Rajan, Kathleen Chang, Sanath Govindarajan, Clara Gulick, Eva English, Bianca Rodriguez, Orion Bloomfield, Stella Nakada, Charlotte Beard, Sarah O’Connor, Sophia Mastroianni, Emma Downey, Matthew Feigenbaum, Caitlin Tolentino, Abigail Pace, Marina Khan, Soyoun Moon, Jordan DiPrima, Amber Syed, Flora Lin, Yasmina Abukhadra, Isabella Bacon, John Beckerle, Sophia Cho, Nana Esi Donkor, Lucy Garberg, Ava Harrington, Mai Hoang, Nosa Lawani, Ayush Noori, Euwie Park, Ella Parsons, Philip Oravitan, Matthew Chen, Cristian Molina, Caleb Richmond, Adith Reddi, Jason Huang, Cooper Shugrue, Rose Coviello, Selma Unver, Matthew Indelicarto, Emir Islamovic, Rosemary McIlroy, Alana Yang, Mahdi Hamad, Elizabeth Griffin, Zara Ahmed, Asha Alla, Patricia Fitzgerald, Audrey Choi, Tanya Das, Yuchen Cheng, Joshua Yu, Tabor Roderiques, Ethan Lee, Longchao Liu, Jaekeb Harper, Jason Wang, Chris Suhr, Max Tan, Jacqueline Luque, A Russell Tam, Emma Chen, Max Triff, Lyric Zimmermann, Eric Zhang, Jackie Wood, Kaitlin Clark, Nat Kpodonu, Antar Dey, Alexander Ecker, Maximilian Chuang, Ramón Kodi Suzuki López, Harry Sun, Zijing Wei, Henry Stone, Chia Yu Joy Chi, Aiden Silvestri, Petra Orloff, Neha Nedumaran, Aletheia Zou, Leyla Ünver, Oscair Page, Minseo Kim, Terence Yan Tao Chan, Akili Tulloch, Andrea Hernandez, Aruli Pillai, Caitlyn Chen, Neil Chowdhury, Lina Huang, Anish Mudide, Garrett Paik, Alexandra Wingate, Lily Quinn, Chris Conybere, Luca Laiza Baumgardt, Rollo Buckley, Zara Kolberg, Ruth Pattison, Ashlyn Ahmad Shazli, Pia Ganske, Luca Sfragara, Annina Strub, Barney Collier, Hari Tamana, Dylan Ravindran, James Howden, Madeleine Stewart, Sakura Shimizu, Julia Braniff, Melanie Fong, Lucy Gutman, Danny Irvine, Sahil Malholtra, Jillian Medina, John Park, Alicia Yin, Harrison Abromavage, Breanna Barrett, Jacqueline Chen, Rachelle Cho, Mac Dilatush, Gabriel Gaw, Caitlin Gu, Jupiter Huang, Houston Kilby, Ethan Markel, Katie McClure, William Phillips, Benjamin Polaski, Amelia Roselli, Soleil Saint-Cyr, Ellie Shin, Kylan Tatum, Tai Tumpunyawat, Lucia Wetherill, Sara Ptaszynska, Maddie Zeleznik, Alexander Pesendorfer, Anna Nolan, Jeffrey Tao, Divya Sammeta, Laney Nicholson, Giao Vu Dinh, Merrin Foltz, An Vo, Maggie Ross, Andrew Tokarski, Samika Hariharan, Elaine Wang, Martha Baziuk, Ashley Tay, Yuk Hung Maximus Wong, Jax Floyd, Aileen Cui, Kieran Pierre, Nikita Coppisetti, Matthew Kutam, Dhruv Khurjekar, Anthony Gadzi, Ben Gubbay, Sophia Pedretti, Sofiya Belovich, Tiffany Yeung, Mercy Fey, Layla Shaffer, Arthur Li, Giancarlo Beritela, Kyle Huyghue, Greg Foster, Garrett Durso-Finley, Quinn Thierfelder, Holly Kiernan, Andrew Lenkowsky, Tesia Thomas, Nicole Cheng, Olivia Chao, Pia L’Etoile-Goga, Alexa King, Paris McKinley, Nicole Read, David Milberg, Leila Lin, Melinda Wong, Io Gilman, Samantha Brown, Lila Chen, Jordyn Kosai, Mark Verbinsky, Alice Belshaw-Hood, Honon Lee, Cathy Zhou, Maya Lobo, Asia Tse, Kyle Tran, Kira Lewis, Pratmesh Sonawane, Jonathan Ngo, Sophia Zuzga, Lillian Chow, Vianne Huynh, Wenyi Yang, Samantha Lim, Brandon Stites, Shannon Chang, Raenalyn Cruz-Balleza, Michaela Pelta, Stella Kujawski, Christopher Yuan, Elio Standen-Bloom, Oliver Witt, Karina Anders, Audrey Duane, Nancy Huynh, Benjamin Lester, Samantha Fung-Lee, Melanie Fung, Mandy Situ, Paolo Canigiula, Matijs Dijkgraaf, Wilbert Romero, Samantha Karmela Baula, Kimberly Wong, Ivana Xu, Benjamin Martinez, Reena Nuygen, Lucy Norris, Noah Nijensohn, Naomi Altman, Elise Maajid, Olivia Burkhardt, Jullian Chanda, Catherine Doscher, Alex Gopal, Aaron Good, Jonah Good, Nate Herrera, Lucas Lanting, Sophia Liem, Anila Marks, Emma McLaughlin, Audrey Lee, Collin Mohr, Emma Patton, Naima Pyarali, Claire Oczon, Daniel Richards, Nathan Good, Spencer Goss, Adeeb Khan, Reagan Madonia, Vivian Mitchell, Natasha Sun, Tarik Vranka, Diogo Garcia, Frida Arroyo, Eric Morales, Steven Camey, Giovanni Cano, Angelica Bernabe, Jennifer Arroyo, Yadira Lopez, Emily Gonzalez, Bryan Zumba, Josue Garcia, Esmeralda Vargas, Allen Trinidad, Noel Candelaria, Vanessa Valdez, Faith Campuzano, Emily Pereznegron, Jenifer Medrano, Jonathan Gutierrez, Evelyn Gutierrez, Ericka Taboada Abrego, Dayanara Gutierrez, Cristian Ortiz, Angelica Barnes, Eleanor Arms, Leo Mitchell, Ciara Balanzá, Jake Bradford, Harrison Detroy, Devin Ferguson, Ethel Guillermo, Anusha Manapragada, Daniella Nanula, Brigitte Serna, Khushi Singh, Emily Sramaty, Brian Wells, Matthew Wiggins, Melissa Dowling, Geraldine Schmadeke, Samantha Cafferky, Stephanie Good, Margaret Reese, Miranda Fleig, Alex Gannett, Cory Cain, Melody Lee, Paul Oberto, Jennifer Rinehart, Elaine Pan, Sallie Anne Mathis, Jessica Joiner, Leslie Barr, Cory J Evans, Alberto Baena-Lopez, Andrea Beatty, Jeanette Collette, Robert Smullen, Jeanne Suttie, Townley Chisholm, Cheryl Rotondo, Gareth Lewis, Victoria Turner, Lloyd Stark, Elizabeth Fox, Anjana Amirapu, Sangbin Park, Nicole Lantz, Anne E Rankin, Seung K Kim, Lutz Kockel

**Affiliations:** Phillips Exeter Academy, Exeter, NH 03833, USA; Department of Developmental Biology, Stanford University School of Medicine, Stanford, CA 94305, USA; Department of Developmental Biology, Stanford University School of Medicine, Stanford, CA 94305, USA; Phillips Exeter Academy, Exeter, NH 03833, USA; Phillips Exeter Academy, Exeter, NH 03833, USA; Phillips Exeter Academy, Exeter, NH 03833, USA; Pritzker College Prep, Chicago, IL 60639, USA; Phillips Exeter Academy, Exeter, NH 03833, USA; Dalton School, New York, NY 10128, USA; Haileybury School, Hertford SG13 7NU, UK; Commack High School, 1 Scholar Ln, Commack, NY 11725, USA; Commack High School, 1 Scholar Ln, Commack, NY 11725, USA; Commack High School, 1 Scholar Ln, Commack, NY 11725, USA; Commack High School, 1 Scholar Ln, Commack, NY 11725, USA; Commack High School, 1 Scholar Ln, Commack, NY 11725, USA; Commack High School, 1 Scholar Ln, Commack, NY 11725, USA; Commack High School, 1 Scholar Ln, Commack, NY 11725, USA; Commack High School, 1 Scholar Ln, Commack, NY 11725, USA; Commack High School, 1 Scholar Ln, Commack, NY 11725, USA; Commack High School, 1 Scholar Ln, Commack, NY 11725, USA; Commack High School, 1 Scholar Ln, Commack, NY 11725, USA; Phillips Exeter Academy, Exeter, NH 03833, USA; Phillips Exeter Academy, Exeter, NH 03833, USA; Phillips Exeter Academy, Exeter, NH 03833, USA; Phillips Exeter Academy, Exeter, NH 03833, USA; Phillips Exeter Academy, Exeter, NH 03833, USA; Phillips Exeter Academy, Exeter, NH 03833, USA; Phillips Exeter Academy, Exeter, NH 03833, USA; Phillips Exeter Academy, Exeter, NH 03833, USA; Phillips Exeter Academy, Exeter, NH 03833, USA; Phillips Exeter Academy, Exeter, NH 03833, USA; Phillips Exeter Academy, Exeter, NH 03833, USA; Phillips Exeter Academy, Exeter, NH 03833, USA; Phillips Exeter Academy, Exeter, NH 03833, USA; Phillips Exeter Academy, Exeter, NH 03833, USA; Phillips Exeter Academy, Exeter, NH 03833, USA; Phillips Exeter Academy, Exeter, NH 03833, USA; Phillips Exeter Academy, Exeter, NH 03833, USA; Phillips Exeter Academy, Exeter, NH 03833, USA; Phillips Exeter Academy, Exeter, NH 03833, USA; Phillips Exeter Academy, Exeter, NH 03833, USA; Phillips Exeter Academy, Exeter, NH 03833, USA; Phillips Exeter Academy, Exeter, NH 03833, USA; Phillips Exeter Academy, Exeter, NH 03833, USA; Phillips Exeter Academy, Exeter, NH 03833, USA; Phillips Exeter Academy, Exeter, NH 03833, USA; Phillips Exeter Academy, Exeter, NH 03833, USA; Phillips Exeter Academy, Exeter, NH 03833, USA; Phillips Exeter Academy, Exeter, NH 03833, USA; Phillips Exeter Academy, Exeter, NH 03833, USA; Phillips Exeter Academy, Exeter, NH 03833, USA; Phillips Exeter Academy, Exeter, NH 03833, USA; Phillips Exeter Academy, Exeter, NH 03833, USA; Phillips Exeter Academy, Exeter, NH 03833, USA; Phillips Exeter Academy, Exeter, NH 03833, USA; Phillips Exeter Academy, Exeter, NH 03833, USA; Phillips Exeter Academy, Exeter, NH 03833, USA; Phillips Exeter Academy, Exeter, NH 03833, USA; Phillips Exeter Academy, Exeter, NH 03833, USA; Phillips Exeter Academy, Exeter, NH 03833, USA; Phillips Exeter Academy, Exeter, NH 03833, USA; Phillips Exeter Academy, Exeter, NH 03833, USA; Phillips Exeter Academy, Exeter, NH 03833, USA; Phillips Exeter Academy, Exeter, NH 03833, USA; Phillips Exeter Academy, Exeter, NH 03833, USA; Phillips Exeter Academy, Exeter, NH 03833, USA; Phillips Exeter Academy, Exeter, NH 03833, USA; Phillips Exeter Academy, Exeter, NH 03833, USA; Phillips Exeter Academy, Exeter, NH 03833, USA; Phillips Exeter Academy, Exeter, NH 03833, USA; Phillips Exeter Academy, Exeter, NH 03833, USA; Phillips Exeter Academy, Exeter, NH 03833, USA; Phillips Exeter Academy, Exeter, NH 03833, USA; Phillips Exeter Academy, Exeter, NH 03833, USA; Phillips Exeter Academy, Exeter, NH 03833, USA; Phillips Exeter Academy, Exeter, NH 03833, USA; Phillips Exeter Academy, Exeter, NH 03833, USA; Phillips Exeter Academy, Exeter, NH 03833, USA; Phillips Exeter Academy, Exeter, NH 03833, USA; Phillips Exeter Academy, Exeter, NH 03833, USA; Phillips Exeter Academy, Exeter, NH 03833, USA; Phillips Exeter Academy, Exeter, NH 03833, USA; Phillips Exeter Academy, Exeter, NH 03833, USA; Phillips Exeter Academy, Exeter, NH 03833, USA; Phillips Exeter Academy, Exeter, NH 03833, USA; Phillips Exeter Academy, Exeter, NH 03833, USA; Phillips Exeter Academy, Exeter, NH 03833, USA; Phillips Exeter Academy, Exeter, NH 03833, USA; Phillips Exeter Academy, Exeter, NH 03833, USA; Phillips Exeter Academy, Exeter, NH 03833, USA; Phillips Exeter Academy, Exeter, NH 03833, USA; Phillips Exeter Academy, Exeter, NH 03833, USA; Phillips Exeter Academy, Exeter, NH 03833, USA; Phillips Exeter Academy, Exeter, NH 03833, USA; Phillips Exeter Academy, Exeter, NH 03833, USA; Phillips Exeter Academy, Exeter, NH 03833, USA; Haileybury School, Hertford SG13 7NU, UK; Haileybury School, Hertford SG13 7NU, UK; Haileybury School, Hertford SG13 7NU, UK; Haileybury School, Hertford SG13 7NU, UK; Haileybury School, Hertford SG13 7NU, UK; Haileybury School, Hertford SG13 7NU, UK; Haileybury School, Hertford SG13 7NU, UK; Haileybury School, Hertford SG13 7NU, UK; Haileybury School, Hertford SG13 7NU, UK; Haileybury School, Hertford SG13 7NU, UK; Haileybury School, Hertford SG13 7NU, UK; Haileybury School, Hertford SG13 7NU, UK; Haileybury School, Hertford SG13 7NU, UK; Haileybury School, Hertford SG13 7NU, UK; Haileybury School, Hertford SG13 7NU, UK; Haileybury School, Hertford SG13 7NU, UK; The Lawrenceville School, 2500 Main St, Lawrenceville, NJ 08648, USA; The Lawrenceville School, 2500 Main St, Lawrenceville, NJ 08648, USA; The Lawrenceville School, 2500 Main St, Lawrenceville, NJ 08648, USA; The Lawrenceville School, 2500 Main St, Lawrenceville, NJ 08648, USA; The Lawrenceville School, 2500 Main St, Lawrenceville, NJ 08648, USA; The Lawrenceville School, 2500 Main St, Lawrenceville, NJ 08648, USA; The Lawrenceville School, 2500 Main St, Lawrenceville, NJ 08648, USA; The Lawrenceville School, 2500 Main St, Lawrenceville, NJ 08648, USA; The Lawrenceville School, 2500 Main St, Lawrenceville, NJ 08648, USA; The Lawrenceville School, 2500 Main St, Lawrenceville, NJ 08648, USA; The Lawrenceville School, 2500 Main St, Lawrenceville, NJ 08648, USA; The Lawrenceville School, 2500 Main St, Lawrenceville, NJ 08648, USA; The Lawrenceville School, 2500 Main St, Lawrenceville, NJ 08648, USA; The Lawrenceville School, 2500 Main St, Lawrenceville, NJ 08648, USA; The Lawrenceville School, 2500 Main St, Lawrenceville, NJ 08648, USA; The Lawrenceville School, 2500 Main St, Lawrenceville, NJ 08648, USA; The Lawrenceville School, 2500 Main St, Lawrenceville, NJ 08648, USA; The Lawrenceville School, 2500 Main St, Lawrenceville, NJ 08648, USA; The Lawrenceville School, 2500 Main St, Lawrenceville, NJ 08648, USA; The Lawrenceville School, 2500 Main St, Lawrenceville, NJ 08648, USA; The Lawrenceville School, 2500 Main St, Lawrenceville, NJ 08648, USA; The Lawrenceville School, 2500 Main St, Lawrenceville, NJ 08648, USA; The Lawrenceville School, 2500 Main St, Lawrenceville, NJ 08648, USA; The Lawrenceville School, 2500 Main St, Lawrenceville, NJ 08648, USA; The Lawrenceville School, 2500 Main St, Lawrenceville, NJ 08648, USA; The Lawrenceville School, 2500 Main St, Lawrenceville, NJ 08648, USA; The Lawrenceville School, 2500 Main St, Lawrenceville, NJ 08648, USA; The Lawrenceville School, 2500 Main St, Lawrenceville, NJ 08648, USA; The Lawrenceville School, 2500 Main St, Lawrenceville, NJ 08648, USA; The Lawrenceville School, 2500 Main St, Lawrenceville, NJ 08648, USA; The Lawrenceville School, 2500 Main St, Lawrenceville, NJ 08648, USA; The Lawrenceville School, 2500 Main St, Lawrenceville, NJ 08648, USA; The Lawrenceville School, 2500 Main St, Lawrenceville, NJ 08648, USA; The Lawrenceville School, 2500 Main St, Lawrenceville, NJ 08648, USA; The Lawrenceville School, 2500 Main St, Lawrenceville, NJ 08648, USA; The Lawrenceville School, 2500 Main St, Lawrenceville, NJ 08648, USA; The Lawrenceville School, 2500 Main St, Lawrenceville, NJ 08648, USA; The Lawrenceville School, 2500 Main St, Lawrenceville, NJ 08648, USA; The Lawrenceville School, 2500 Main St, Lawrenceville, NJ 08648, USA; The Lawrenceville School, 2500 Main St, Lawrenceville, NJ 08648, USA; The Lawrenceville School, 2500 Main St, Lawrenceville, NJ 08648, USA; The Lawrenceville School, 2500 Main St, Lawrenceville, NJ 08648, USA; The Lawrenceville School, 2500 Main St, Lawrenceville, NJ 08648, USA; The Lawrenceville School, 2500 Main St, Lawrenceville, NJ 08648, USA; The Lawrenceville School, 2500 Main St, Lawrenceville, NJ 08648, USA; The Lawrenceville School, 2500 Main St, Lawrenceville, NJ 08648, USA; The Lawrenceville School, 2500 Main St, Lawrenceville, NJ 08648, USA; The Lawrenceville School, 2500 Main St, Lawrenceville, NJ 08648, USA; The Lawrenceville School, 2500 Main St, Lawrenceville, NJ 08648, USA; The Lawrenceville School, 2500 Main St, Lawrenceville, NJ 08648, USA; The Lawrenceville School, 2500 Main St, Lawrenceville, NJ 08648, USA; The Lawrenceville School, 2500 Main St, Lawrenceville, NJ 08648, USA; The Lawrenceville School, 2500 Main St, Lawrenceville, NJ 08648, USA; The Lawrenceville School, 2500 Main St, Lawrenceville, NJ 08648, USA; The Lawrenceville School, 2500 Main St, Lawrenceville, NJ 08648, USA; The Lawrenceville School, 2500 Main St, Lawrenceville, NJ 08648, USA; The Lawrenceville School, 2500 Main St, Lawrenceville, NJ 08648, USA; The Lawrenceville School, 2500 Main St, Lawrenceville, NJ 08648, USA; The Lawrenceville School, 2500 Main St, Lawrenceville, NJ 08648, USA; The Lawrenceville School, 2500 Main St, Lawrenceville, NJ 08648, USA; The Lawrenceville School, 2500 Main St, Lawrenceville, NJ 08648, USA; The Lawrenceville School, 2500 Main St, Lawrenceville, NJ 08648, USA; The Lawrenceville School, 2500 Main St, Lawrenceville, NJ 08648, USA; The Lawrenceville School, 2500 Main St, Lawrenceville, NJ 08648, USA; The Lawrenceville School, 2500 Main St, Lawrenceville, NJ 08648, USA; The Lawrenceville School, 2500 Main St, Lawrenceville, NJ 08648, USA; The Lawrenceville School, 2500 Main St, Lawrenceville, NJ 08648, USA; Lowell High School, 1101 Eucalyptus Dr, San Francisco, CA 94132, USA; Lowell High School, 1101 Eucalyptus Dr, San Francisco, CA 94132, USA; Lowell High School, 1101 Eucalyptus Dr, San Francisco, CA 94132, USA; Lowell High School, 1101 Eucalyptus Dr, San Francisco, CA 94132, USA; Lowell High School, 1101 Eucalyptus Dr, San Francisco, CA 94132, USA; Lowell High School, 1101 Eucalyptus Dr, San Francisco, CA 94132, USA; Lowell High School, 1101 Eucalyptus Dr, San Francisco, CA 94132, USA; Lowell High School, 1101 Eucalyptus Dr, San Francisco, CA 94132, USA; Lowell High School, 1101 Eucalyptus Dr, San Francisco, CA 94132, USA; Lowell High School, 1101 Eucalyptus Dr, San Francisco, CA 94132, USA; Lowell High School, 1101 Eucalyptus Dr, San Francisco, CA 94132, USA; Lowell High School, 1101 Eucalyptus Dr, San Francisco, CA 94132, USA; Lowell High School, 1101 Eucalyptus Dr, San Francisco, CA 94132, USA; Lowell High School, 1101 Eucalyptus Dr, San Francisco, CA 94132, USA; Lowell High School, 1101 Eucalyptus Dr, San Francisco, CA 94132, USA; Lowell High School, 1101 Eucalyptus Dr, San Francisco, CA 94132, USA; Lowell High School, 1101 Eucalyptus Dr, San Francisco, CA 94132, USA; Lowell High School, 1101 Eucalyptus Dr, San Francisco, CA 94132, USA; Lowell High School, 1101 Eucalyptus Dr, San Francisco, CA 94132, USA; Lowell High School, 1101 Eucalyptus Dr, San Francisco, CA 94132, USA; Lowell High School, 1101 Eucalyptus Dr, San Francisco, CA 94132, USA; Lowell High School, 1101 Eucalyptus Dr, San Francisco, CA 94132, USA; Lowell High School, 1101 Eucalyptus Dr, San Francisco, CA 94132, USA; Lowell High School, 1101 Eucalyptus Dr, San Francisco, CA 94132, USA; Lowell High School, 1101 Eucalyptus Dr, San Francisco, CA 94132, USA; Lowell High School, 1101 Eucalyptus Dr, San Francisco, CA 94132, USA; Lowell High School, 1101 Eucalyptus Dr, San Francisco, CA 94132, USA; Lowell High School, 1101 Eucalyptus Dr, San Francisco, CA 94132, USA; Lowell High School, 1101 Eucalyptus Dr, San Francisco, CA 94132, USA; Lowell High School, 1101 Eucalyptus Dr, San Francisco, CA 94132, USA; Lowell High School, 1101 Eucalyptus Dr, San Francisco, CA 94132, USA; Lowell High School, 1101 Eucalyptus Dr, San Francisco, CA 94132, USA; Lowell High School, 1101 Eucalyptus Dr, San Francisco, CA 94132, USA; Lowell High School, 1101 Eucalyptus Dr, San Francisco, CA 94132, USA; Lowell High School, 1101 Eucalyptus Dr, San Francisco, CA 94132, USA; Lowell High School, 1101 Eucalyptus Dr, San Francisco, CA 94132, USA; Lowell High School, 1101 Eucalyptus Dr, San Francisco, CA 94132, USA; Lowell High School, 1101 Eucalyptus Dr, San Francisco, CA 94132, USA; Lowell High School, 1101 Eucalyptus Dr, San Francisco, CA 94132, USA; Lowell High School, 1101 Eucalyptus Dr, San Francisco, CA 94132, USA; Lowell High School, 1101 Eucalyptus Dr, San Francisco, CA 94132, USA; Lowell High School, 1101 Eucalyptus Dr, San Francisco, CA 94132, USA; Lowell High School, 1101 Eucalyptus Dr, San Francisco, CA 94132, USA; Lowell High School, 1101 Eucalyptus Dr, San Francisco, CA 94132, USA; Lowell High School, 1101 Eucalyptus Dr, San Francisco, CA 94132, USA; Lowell High School, 1101 Eucalyptus Dr, San Francisco, CA 94132, USA; Lowell High School, 1101 Eucalyptus Dr, San Francisco, CA 94132, USA; Lowell High School, 1101 Eucalyptus Dr, San Francisco, CA 94132, USA; Latin School of Chicago, 59 W North Blvd, Chicago, IL 60610, USA; Latin School of Chicago, 59 W North Blvd, Chicago, IL 60610, USA; Latin School of Chicago, 59 W North Blvd, Chicago, IL 60610, USA; Latin School of Chicago, 59 W North Blvd, Chicago, IL 60610, USA; Latin School of Chicago, 59 W North Blvd, Chicago, IL 60610, USA; Latin School of Chicago, 59 W North Blvd, Chicago, IL 60610, USA; Albuquerque Academy, Albuquerque, NM 87109, USA; Albuquerque Academy, Albuquerque, NM 87109, USA; Albuquerque Academy, Albuquerque, NM 87109, USA; Albuquerque Academy, Albuquerque, NM 87109, USA; Albuquerque Academy, Albuquerque, NM 87109, USA; Albuquerque Academy, Albuquerque, NM 87109, USA; Albuquerque Academy, Albuquerque, NM 87109, USA; Albuquerque Academy, Albuquerque, NM 87109, USA; Albuquerque Academy, Albuquerque, NM 87109, USA; Albuquerque Academy, Albuquerque, NM 87109, USA; Albuquerque Academy, Albuquerque, NM 87109, USA; Albuquerque Academy, Albuquerque, NM 87109, USA; Albuquerque Academy, Albuquerque, NM 87109, USA; Albuquerque Academy, Albuquerque, NM 87109, USA; Albuquerque Academy, Albuquerque, NM 87109, USA; Albuquerque Academy, Albuquerque, NM 87109, USA; Albuquerque Academy, Albuquerque, NM 87109, USA; Albuquerque Academy, Albuquerque, NM 87109, USA; Albuquerque Academy, Albuquerque, NM 87109, USA; Albuquerque Academy, Albuquerque, NM 87109, USA; Albuquerque Academy, Albuquerque, NM 87109, USA; Albuquerque Academy, Albuquerque, NM 87109, USA; Albuquerque Academy, Albuquerque, NM 87109, USA; Albuquerque Academy, Albuquerque, NM 87109, USA; Pritzker College Prep, Chicago, IL 60639, USA; Pritzker College Prep, Chicago, IL 60639, USA; Pritzker College Prep, Chicago, IL 60639, USA; Pritzker College Prep, Chicago, IL 60639, USA; Pritzker College Prep, Chicago, IL 60639, USA; Pritzker College Prep, Chicago, IL 60639, USA; Pritzker College Prep, Chicago, IL 60639, USA; Pritzker College Prep, Chicago, IL 60639, USA; Pritzker College Prep, Chicago, IL 60639, USA; Pritzker College Prep, Chicago, IL 60639, USA; Pritzker College Prep, Chicago, IL 60639, USA; Pritzker College Prep, Chicago, IL 60639, USA; Pritzker College Prep, Chicago, IL 60639, USA; Pritzker College Prep, Chicago, IL 60639, USA; Pritzker College Prep, Chicago, IL 60639, USA; Pritzker College Prep, Chicago, IL 60639, USA; Pritzker College Prep, Chicago, IL 60639, USA; Pritzker College Prep, Chicago, IL 60639, USA; Pritzker College Prep, Chicago, IL 60639, USA; Pritzker College Prep, Chicago, IL 60639, USA; Pritzker College Prep, Chicago, IL 60639, USA; Pritzker College Prep, Chicago, IL 60639, USA; Pritzker College Prep, Chicago, IL 60639, USA; Chapin School, New York, NY 10028, USA; Chapin School, New York, NY 10028, USA; Dalton School, New York, NY 10128, USA; Dalton School, New York, NY 10128, USA; Loyola Marymount University, Los Angeles, CA 90045, USA; Loyola Marymount University, Los Angeles, CA 90045, USA; Loyola Marymount University, Los Angeles, CA 90045, USA; Loyola Marymount University, Los Angeles, CA 90045, USA; Loyola Marymount University, Los Angeles, CA 90045, USA; Loyola Marymount University, Los Angeles, CA 90045, USA; Loyola Marymount University, Los Angeles, CA 90045, USA; Loyola Marymount University, Los Angeles, CA 90045, USA; Loyola Marymount University, Los Angeles, CA 90045, USA; Loyola Marymount University, Los Angeles, CA 90045, USA; Loyola Marymount University, Los Angeles, CA 90045, USA; Latin School of Chicago, 59 W North Blvd, Chicago, IL 60610, USA; Latin School of Chicago, 59 W North Blvd, Chicago, IL 60610, USA; Albuquerque Academy, Albuquerque, NM 87109, USA; Albuquerque Academy, Albuquerque, NM 87109, USA; Albuquerque Academy, Albuquerque, NM 87109, USA; Albuquerque Academy, Albuquerque, NM 87109, USA; Pritzker College Prep, Chicago, IL 60639, USA; Pritzker College Prep, Chicago, IL 60639, USA; Harvard-Westlake School, Los Angeles, CA 90077, USA; Hotchkiss School, Lakeville, CT 06039, USA; Hotchkiss School, Lakeville, CT 06039, USA; Chapin School, New York, NY 10028, USA; Chapin School, New York, NY 10028, USA; Dalton School, New York, NY 10128, USA; Westtown School, West Chester, PA 19382, USA; Loyola Marymount University, Los Angeles, CA 90045, USA; University of Oxford, Oxford OX1 3RE, UK; Commack High School, 1 Scholar Ln, Commack, NY 11725, USA; Commack High School, 1 Scholar Ln, Commack, NY 11725, USA; Commack High School, 1 Scholar Ln, Commack, NY 11725, USA; Commack High School, 1 Scholar Ln, Commack, NY 11725, USA; Phillips Exeter Academy, Exeter, NH 03833, USA; Phillips Exeter Academy, Exeter, NH 03833, USA; Haileybury School, Hertford SG13 7NU, UK; Haileybury School, Hertford SG13 7NU, UK; Haileybury School, Hertford SG13 7NU, UK; The Lawrenceville School, 2500 Main St, Lawrenceville, NJ 08648, USA; Lowell High School, 1101 Eucalyptus Dr, San Francisco, CA 94132, USA; Department of Developmental Biology, Stanford University School of Medicine, Stanford, CA 94305, USA; The Lawrenceville School, 2500 Main St, Lawrenceville, NJ 08648, USA; Phillips Exeter Academy, Exeter, NH 03833, USA; Department of Developmental Biology, Stanford University School of Medicine, Stanford, CA 94305, USA; Department of Developmental Biology, Stanford University School of Medicine, Stanford, CA 94305, USA

**Keywords:** LexA, *patched (ptc)*, natural transposons, insulin producing cells, L3 brain, enhancer trap, high school biology class, Stan-X, 1360, Copia

## Abstract

Conditional gene regulation in *Drosophila* through binary expression systems like the LexA-LexAop system provides a superb tool for investigating gene and tissue function. To increase the availability of defined LexA enhancer trap insertions, we present molecular, genetic, and tissue expression studies of 301 novel Stan-X LexA enhancer traps derived from mobilization of the index SX4 line. This includes insertions into distinct loci on the X, II, and III chromosomes that were not previously associated with enhancer traps or targeted LexA constructs, an insertion into *ptc*, and seventeen insertions into natural transposons. A subset of enhancer traps was expressed in CNS neurons known to produce and secrete insulin, an essential regulator of growth, development, and metabolism. Fly lines described here were generated and characterized through studies by students and teachers in an international network of genetics classes at public, independent high schools, and universities serving a diversity of students, including those underrepresented in science. Thus, a unique partnership between secondary schools and university-based programs has produced and characterized novel resources in *Drosophila*, establishing instructional paradigms devoted to unscripted experimental science.

## Introduction

Conditional gene expression systems in *Drosophila* provide a powerful basis for investigating the function and regulation of genes and cells. Generation of a GAL4-based transactivator to induce expression of target genes fused to upstream activating sequences (UAS) is a widely used binary expression system in *Drosophila* (Brand and Perrimon 1993; Hayashi *et al*. 2002; Gohl *et al*. 2011). Random insertions by transposons encoding GAL4 into the genome (“enhancer trapping”; O’Kane and Gehring 1987) generate strains with endogenous enhancer-directed GAL4 expression. In these enhancer trapping constructs, a weak promoter “reads” the local enhancer landscape and directs the expression of Gal4 according to this regulatory information (Sepp and Auld 1999). Studies of many biological problems benefit from simultaneous manipulation of two or more independent cell populations or genes (reviewed in Rajan and Perrimon (2012)Kim *et al*. (2021)). In prior studies, parallel use of two binary expression systems allowed important new biological insights, including clonal and lineage analysis (Lai and Lee 2006; Bosch *et al*. 2015), “tissue epistasis” studies (Yagi *et al*. 2010; Shim *et al*. 2013), and discovery of specific cell–cell interactions and contacts (Gordon and Scott 2009; Bosch *et al*. 2015; Macpherson *et al*. 2015). These approaches used a second expression system that functions independently of the UAS-Gal4 system, such as the LexA system derived from a bacterial DNA-binding domain (Szüts and Bienz 2000; Lai and Lee 2006; Pfeiffer *et al*. 2010; Gnerer *et al*. 2015; Knapp *et al*. 2015). The fusion of the LexA DNA-binding domain to a transactivator domain generates a protein that regulates expression of transgenes linked to a LexA operator–promoter (LexAop). However, the number and quality of lines expressing a LexA transactivator remain small, compared to the thousands of comparable GAL4-based lines.

To address this resource gap, we previously developed a network of partnerships between a research university (Stanford) and US secondary schools to generate novel LexA-based enhancer trap drivers, in an outreach we called “Stan-X” (Kockel *et al*. 2016, 2019, https://www.stan-x.org/). In these high schools, the enhancer trap and molecular biology experiments whose results are outlined and documented below are integrated into the class schedules as an advanced biology course (colloquially referred to as a “Stan-X course”). This class was taught by instructors during the regular school year, with students receiving educational credit.

Here, we describe a significant expansion of this earlier effort into an international scholastic network including Stanford University, and science classes at seventeen independent and public secondary schools and universities in the United States and United Kingdom. To increase our capacity for training the participating high school teachers, we established a teacher training academy, called “Discovery Now”. Over 2 weeks each summer, incoming and participating teachers are instructed in underlying principles of genetics and molecular biology, followed by weekly meetings during the subsequent school year. This expanded network of participating high schools successfully produced hundreds of novel LexA-based enhancer-trap lines for the community of science, advancing science instruction paradigms rooted in experimental genetics, molecular, and cell biology.

## Materials and methods

### Construction of the SX4 LexA enhancer-trap element

The SX4 P-element carries a LexA::G4 fusion (LexA DNA-binding domain, “L”, the Gal4 hinge region, “H”, and the Gal4 transcriptional activation domain, “G”, construct “LHG”) identical to the SE1 P-element (Kockel *et al*. 2016), under the control of the hsp70 promoter. The 3,563 bp EagI–EagI fragment from pDPPattB-LHG (Yagi *et al*. 2010) was subcloned to the 7,097 bp EagI–EagI fragment from pJFRC-MUH (Pfeiffer *et al*. 2010) to make pJFRC-MUH-70LHG70 (construct #1). The 3,615 bp NotI–NotI fragment from pXN-attPGAL4LWL (Gohl *et al*. 2011) was subcloned to the NotI site on pBS2KSP vector to make pBS2KSP-attP-Pprom-GAL4-hsp70 3′UTR (construct #2). The 3,842 bp (NheI)–(EcoRI) fragment from pJFRC-MUH-70LHG70 (construct #1) was Klenow filled-in and ligated to 3,390 bp EcoRV–EcoRV fragment from pBS2KSP-attP-Pprom-GAL4-hsp70 3′UTR (construct #2) to generate pBS2KSP-attP-hsp70TATA-LHG-hsp70 3′UTR (construct #3). The 4,098 bp SacII–XbaI fragment from pBS2KSP-attP-hsp70TATA-LHG-hsp70 3′UTR (construct #3) was subcloned to 8,453 bp SacI–XbaI fragment from pXN-attPGAL4LwL (Gohl *et al*. 2011) to generate pXN-attP-hsp70TATA-LHG-LwL (hereafter called “SX2”).

A 904 bp PCR product was amplified from Sx2 using the primers XN_attP_delta_F (5′-gccgaattcggtaccGAGCGCCGGAGTATAAATAGAGGCGCTTC-3′) and LHG_R1 (5′-GCTCTGCTGACGAAGATCTACGACAATTGGTT-3′). The 1,220 bp KpnI–PmeI fragment (containing the attP site) in StanEx2 was replaced by the 857 bp KpnI–PmeI fragment of the above amplified PCR product to generate pXN-hsp70TATA-LHG-LwL (hereafter, “SX4”).

The annotated primary DNA sequence of SX4 enhancer trap P-element is presented in Supplementary Data File 1.

### Construction of SX4 starter strains

The transformation of the P{w[+mC] = LHG]Stan-X[SX4]} P-element vector into the *w^1118^* fly strain was performed using standard procedures. The SX4 X-linked index transformant was isogenized to the Stan-X background to generate the *w^1118^, SX4; iso#32^II^; iso#32^III^*. SX4 is located at X:19,887,269 in the *amnesiac* locus (Supplementary Table 1). We noted an Invader natural transposable element (TE) insertion 123 bp upstream of Stan-X[SX4] that is not represented in FlyBase rs6 of the genome, and might be specific to the *w^1118^, SX4; iso#32^II^; iso#32^III^* isogenized background used (see “Fly husbandry and isogenized fly strains” below).

The SX4 X-linked insertion was then mobilized using standard procedures (see below) to the third chromosome balancer *TM6B*, to create the *w^1118^; TM6B,SX4^orig^/ftz,e* starter stain for mobilization to the X chromosome (see “P-element mobilization”). The SX4 P-element on *TM6B* is located at 3L:3,250,470 in the gene encoding *lncRNA:CR43626*.

### L3 dissection and immunohistochemistry (IHC)

*Drosophila* larva transitions from larval stage L1 to larval stage L3 by intermittent molting. Prior to pupariation, L3 larvae stop feeding and migrate to a pupariation site on the side of a vial. Wandering L3 larvae were bisected and inverted in PBS, and all tissues were fixed in 4% formaldehyde/PBS for 30 min, permeabelized in 0.2% Triton X-100/PBS for 4 hours, and blocked in 3% BSA/PBS for 1 hour. All antibody stainings were performed in 3% BSA/PBS, incubation of primary and secondary antibodies were O/N. PBS was used for all rinses and washes (3× each for primary and secondary antibody incubation steps). Antibodies used were as follows: Chicken anti-RFP 1:2,000 (Rockland, 600-901-379); Goat anti-GFP 1:3,000 (Rockland, 600-101-215); Donkey anti-Goat Alexa Fluor 488 (Life Technologies, A11055); Donkey anti-Chicken Cy3 (Jackson ImmunoResearch, 703-165-155); and Donkey anti-Mouse Alexa Fluor 594 (Life Technologies, A21203). All secondary antibodies were used at 1:500. Tissues were dissected off the cuticle and were mounted in SlowFade Gold mounting medium with DAPI (Life Technologies, S36938). See Kockel *et al.* (2016) for a detailed protocol.

### Epifluorescent microscopy

Microscopy was performed on a Zeiss AxioImager M2 with Zeiss filter sets 49 (DAPI) and 38HE (Alexa Fluor 488) using the extended focus function. Used compound epifluorescent microscopes for high schools with all required lenses, installation services, and optional training sessions are available for sale from MicoOptics (https://www.micro-optics.com/).

### Fly husbandry and isogenized fly strains

All fly strains were maintained on a standard cornmeal-molasses diet (http://flystocks.bio.indiana.edu/Fly_Work/media-recipes/molassesfood.htm). The following strains were used as follows: *y^1^,w^1118^* (Bloomington #6598), w*; ry^*506*^,*Sb*^*1*^,*P{ry[+t7.2] = Delta2-3}99B/TM6B,Tb*^*1*^ (Bloomington #1798), crossed to the Stan-X isogenic background (*iso#11^X^; iso#32^II^; iso#32^III^]*, resulting in *w^1118^ iso#11^X^; iso#32^II^; ry*^*506*^,*Sb*^*1*^,*P{ry[+t7.2] = Delta2-3}99B/TM6B,Hu*,*Tb*^*1*^, and the balancer strain w^1118^ iso#11^X^; L*/CyO; ftz*,e*/TM6,Hu,Tb^*1*^. The SX4 element was first established as the X-linked index insertion of the SX4 enhancer trap P-element in a standard white background *w^1118^*, producing *w^1118^,P{w[+mC] = LHG]Stan-X[SX4]*. Subsequently, autosomes II and III of the stain were isogenized to *w^1118^, P{w[mC] = LHG]Stan-X[SX4]}; iso#32^II^; iso#32^III^* (see below). The *TM6B,SX4^orig^,H[^1^,Tb*^*1*^ chromosome was generated by transposition of SX4 to *TM6B,Hu^1^,Tb* as described above.

### Isogenization

Viable second and third chromosomes were isolated from the *y^1^,w^1118^* strain (Bloomington #6898) by outcrossing *y^1^,w^1118^* to *w^1118^*; L*/CyO; ftz*,e*/TM6,Hu,Tb^*1*^, single male backcrosses to the balancer strain, and brother–sister intercrosses. Scoring offspring for loss of balancer resulted in w^1118^; L*/CyO; iso#32^III^ and w^1118^; iso#32^II^; ftz*,e*/TM6,Hu,Tb^*1*^. The two strains were combined to *w^1118^; iso#32^II^; iso#32^III^*. To isogenize the index transformant *w^1118^*,*SX4*, located on the X chromosome, to *iso#32^II^; iso#32^III^*, females *w^1118^*,SX4; L*/CyO; ftz*,e*/TM6,Hu,Tb^*1*^ were crossed to *w^1118^; iso#32^II^; iso#32^III^*, and single male offspring *w^1118^*,*SX4; iso#32^II^/CyO; iso#32^III^/TM6,Hu^1^,Tb*^*1*^ was backcrossed to *w^1118^*,SX4; L*/CyO; ftz*,e*/TM6,Hu^1^,Tb^*1*^. Resulting offspring females *w^1118^*,*SX4; iso#32^II^/CyO; iso#32^III^/TM6,Hu^1^,Tb*^*1*^ were crossed to parental single male to establish *w^1118^*,*SX4; iso#32^II^; iso#32^III^*.

The viable X chromosome *w^1118^, iso#11^X^* was isolated by crossing single males *w^1118^* to *y^1^w^a^FM7c* females, and backcrossing single female offspring to corresponding F_0_ males. Derived strains were scored for loss of *FM7c*. The viable and fertile strain *w^1118^,iso#11^X^* was combined with marked chromosomes on II and III to yield *w^1118^,iso#11^X^*; L*/CyO; ftz*,e*/TM6,Hu^1^,Tb^*1*^. This strain was used to isogenize the X chromosome of the transposase source *Δ2-3*, and to balance novel insertions of *SX4* on *II* and *III* (see below).

### P-element mobilization from X to autosomes II and III

- F_0_: Females of donor stock w^1118^,SX4; iso#32^II^, iso#32^III^ were mated to males w^1118^,iso#11^X^; iso#32^II^; ry^506^,Sb^1^,P{ry[+t7.2] = Delta2-3}99B/TM6B,Tb^1^,Hu^1^.
- F_1_: w^1118^,SX4; iso#32^II^; ry^506^,Sb^1^,P{ry[+t7.2] = Delta2-3}99B/iso#32^III^ males were crossed to w^1118^,iso#11^X^; L*/CyO; ftz*,e*/TM6,Tb^1^,Hu^1^ females.
- F_2_: w^+^ males were mated to w^1118^,iso#11^X^; L*/CyO; ftz*,e*/TM6,Tb^1^,Hu^1^.
- F_3_: The insertion line was stably balanced deploying a brother–sister cross of w^+^ animals that contained CyO and TM6B,Hu^1^,Tb^1^, yielding w^1118^,iso#11^X^; CyO/SX4^#^,iso#32^II^; TM6B,Hu^1^,Tb^1^/ftz*,e* for insertions on chromosome II or w^1118^,iso#11^X^; CyO/L*; TM6B,Hu^1^,Tb^1^/SX4^#^,iso#32^III^ for insertions on chromosome III.

### P-element mobilization from autosome III to X chromosome

The mobilization of a SX4 element located on a third chromosome balancer, TM6B,SX4[orig] to the X chromosome was performed as a pilot experiment in a non-isogenized, mixed background.

- F_0_: Females of donor stock w^1118^; TM6B,SX4^orig^,Hu^1^,Tb^1^/ftz*,e* were mated to males y^1^,w^1118^; CyO, PBac{w[+mC] = Delta2-3.Exel}2/amos^Tft^ (Bloomington #8201).
- F_1_: w^1118^; CyO,PBac{w[+mC] = Delta2-3.Exel}2/+; TM6B,SX4^orig^,Hu^1^,Tb^1^/+ males were crossed to FM6/C(1)DX, y*, f[^1^] (Bloomington #784) females.
- F_2_: w^+^ B^+^ non-CyO, non-TM6B males were mated to FM7a (Bloomington #785) females.
- F_3_ and later: All strains showing a white eye phenotype are discarded as insertions on autosomes. This is the easiest to discern in F_4_ non-FM7a males.

#### Insertion site cloning

We applied an inverse PCR (iPCR) approach (Kockel *et al*. 2019), to molecularly clone the insertion sites of Stan-X SX4 P-elements. DNA restriction enzymes used are as follows: Sau3AI (NEB R0169) and HpaII (NEB R0171); ligase used: T4 DNA Ligase (NEB M0202); 5′ end cloning: inverse PCR primer “Plac1” CAC CCA AGG CTC TGC TCC CAC AAT and “Plac4” ACT GTG CGT TAG GTC CTG TTC ATT GTT; sequencing primer 5′ end: “SP1” ACA CAA CCT TTC CTC TCA ACA; 3′ end cloning: primer pair “Anna” CGC AAA GCT AAT TCA TGC AGC and “SP1Berta” ACA CAA CCT TTC CTC TCA ACA AAA GTC GAT GTC TCT TGC CGA; and sequencing primer 3′ end: “SP1” ACA CAA CCT TTC CTC TCA ACA. For insertions where the sequence of one end only could be determined by iPCR, we pursued a gene-specific PCR approach (Ballinger and Benzer 1989) using P-element and gene-specific primers. The 5′ end specific P-element primer “Chris” is: GCA CAC AAC CTT TCC TCT CAA C, sequencing primer 5′ end: “Sp1”; 3′ end specific P-element primer “Dove”: CCA CGG ACA TGC TAA GGG TTA A, sequencing primer 3′ end: “Dove”; and sequence of gene-specific primers is available upon request. The position and identity of natural TEs, and the insertion of the SX4 element within, were determined by iPCR (Supplementary Table 1) and confirmed with the genome sequence of the host genome *w^1118^*, *SX4*; *iso32^[II]^*; *iso#32^[III]^* by TE Mapper (Supplementary Table 2).

### Generation of sequence logos and position frequency matrices

The construction of the SX4 sequence logo was executed as described (Crooks *et al*. 2004; Kockel *et al*. 2019) using http://weblogo.threeplusone.com/. The input sequence motif data is listed in Supplementary Table 1. The 8 bp genomic insertion site sequence is codirectional to the P-element's direction of insertion (Linheiro and Bergman 2008; Kockel *et al*. 2019). If P-elements are inserted 5′–>3′, the strand of insertion was named + (plus), and unprocessed genomic scaffold sequences as present in FlyBase were used to extract the insertion site sequences. If P-elements are inserted 3′–>5′, the strand of insertion is termed − (minus), and the reverse complement of the genomic scaffold sequences was used to extract these insertion site sequences.

### Genome sequencing

Library construction for genomic sequencing of the *w[1118], Stan-X[SX4]; iso#32[II], iso#32[III]* index line was performed separately for males and females, in two replicates each, using standard Illumina protocols. Kits used were as follows: Illumina NGS Kit Illumina DNA Prep, (M) Tagmentation (24 samples, IPB), #20060060, and Nextera DNA CD Indexes (24 indexes, 24 samples) #20018707. Starting material was 500 ng genomic DNA isolated using the Quiagen DNeasy Blood & Tissue Kit (#69504) following the instruction for insect DNA isolation. Samples were tagmented, purified, and amplified for 5 cycles using the following Nextera DNA index adapters: male replicate 1: H503 (i5) and H710 (i7); male replicate 2: H503 (i5) and H705 (i7); female replicate 1: H503 (i5) and H705 (i7); and female replicate 2: H505 (i5) and H705 (i7). PCR fragments were purified using Sample Purification Beads (Agencourt AMPure XP #A63880), eluted into 32 μl Buffer EB (Quiagen #19086) and submitted to GeneWiz (NGS@genewiz.com) and sequenced on an Illumina HiSeq using 2 × 150 bp sequencing, single index. The genome sequence data of *w[1118], SX4; iso#32[II]; iso#32[III]* is available on sequence read archive (SRA) https://www.ncbi.nlm.nih.gov/sra/PRJNA912892 or accession number PRJNA912892.

### Genome sequence data processing and analysis

We used BWA, SAMtools, and freebayes to perform variant calling. Details of the pipeline, along with specific parameters used, are provided in the StanX_tools repository (https://github.com/sanath-2024/StanX_tools).

To use our short-read dataset to find novel, non-reference transposons (Fig. 6 and Supplementary Table 2), we deploy a similar a strategy as Linheiro and Bergman (2012). We used BWA to find reads that align to both, a canonical transposon sequence as well as the FlyBase reference genome. These “split reads” were processed and sorted into groups based on alignment location and orientation. Details are provided in the StanX_tools repository (https://github.com/sanath-2024/StanX_tools). Our TE mapper represents ground-up multithreaded reimplementation in the Rust language, focusing on performance and simplicity.

For reproduction and verification, the sequence data is deposited on SRA (BioProject accession number PRJNA91289), and a complete build pipeline is accessible (https://github.com/sanath-2024/stan_x_paper_prep).

### Analysis of Fly Cell Atlas IPC and CC cell data

Insulin-producing cell (IPC) and corpora cardiaca (CC) cell nuclei isolation from males and females was conducted in the framework of the Fly Cell Atlas (FCA, Li *et al*. 2022, https://www.ebi.ac.uk/biostudies/files/E-MTAB-10628/E-MTAB-10628.sdrf.txt). FASTQ sequencing files were aligned to BDGP6 version of the fly whole genome using HISAT2 (Kim *et al*. 2019). Single cell nuclei RNAseq libraries representing IPCs and CC cells were filtered based on dilp2, dilp3, dilp5, and akh expression, respectively. The location of natural transposons (nTEs) and gene locations in the BDGP6 genome were taken from FlyBase. featureCounts (Liao *et al*. 2019) was used to assign aligned reads to transposons or genes and to obtain a count matrix for each library. When quantifying counts for nTEs, multi-mapped reads were assigned their full value to each alignment, which gives a theoretical upper bound for how much transcript could exist for a single nTE. When quantifying counts for classes of nTEs or for all TE expression (Supplementary Fig. 2C), multi-mapped reads were assigned a value of 1/x to each alignment, where x is the number of alignments, which estimates the total amount of reads associated with the class of TE or the total number of reads coming from TEs. Count matrices were used as input to Seurat (Hao *et al*. 2021). Seurat VlnPlot function was used to plot unnormalized counts for gene expression (Supplementary Fig. 2).

### Training of Stan-X teachers at the Discover Now Teacher Academy

For incoming Stan-X teachers, the Stan-X Biology Course covering P-element mobilization (“Module 1”), insertion site sequencing by iPCR (“Module 2”), and expression analysis in third instar larvae (“Module 3”) was offered as a 2-week training course consisting of a 1-week online (∼3 hours/day) session, followed by a 1 week session of in-person training (8 hours/day) at the Lawrenceville School, NJ, or Stanford University School of Medicine. The 2-week class was offered each year in the summer or winter. The course was staffed by instructors from participating high schools and Stanford University School of Medicine. Application deadlines and other information are detailed online at https://www.stan-x.org/.

### High school coursework

All three Stan-X Biology Course modules are taught at Phillips Exeter Academy, NH; Commack High School, Dalton School, and Chapin School in NY; Pritzker College Prep and Latin School of Chicago, both in Chicago, IL; The Lawrenceville School, NJ; Lowell High School, San Francisco, CA; Loyola Marymount University and Harvard-Westlake School, both in Los Angeles, CA; Albuquerque Academy in Albuquerque, NM; Haileybury, Hertford, UK; Westtown School, West Chester, PA; and the Hotchkiss School, Lakeville, CT, and Harvard University, Division of Continuing Education (ECPS). Students at individual schools are selected by individual schools for the Stan-X course by teachers at each respective school.

Secondary school students spent 9–10 weeks executing the P-element mobilization crosses, mapping and balancing their novel SX4 strains. This was followed by 2–3 weeks for molecular determination of the SX4 insertion site, using iPCR and DNA sequencing using spin column-based genomic DNA preparation. The last weeks of classes are reserved for crosses with reporter strains (*w; LexAop2-CD8::GFP*), allowing for training in L3 larval dissection and epifluorescent microscopy to describe tissue specific expression patterns of novel SX4 enhancer traps.

Based on performance and recommendation of Stan-X teachers, one to three students were invited to continue studies at Stanford University School of Medicine during summer internships lasting from 2–6 weeks. These studies included further molecular mapping of transposon insertion sites and verification of tissue patterns of enhancer trap expression. Students returning in the fall term helped instructors to run the subsequent iteration of the Stan-X class, and also pursue independent projects.

## Results

### Generation of starter fly lines for LexA enhancer trap screening

**Fig. 1.**
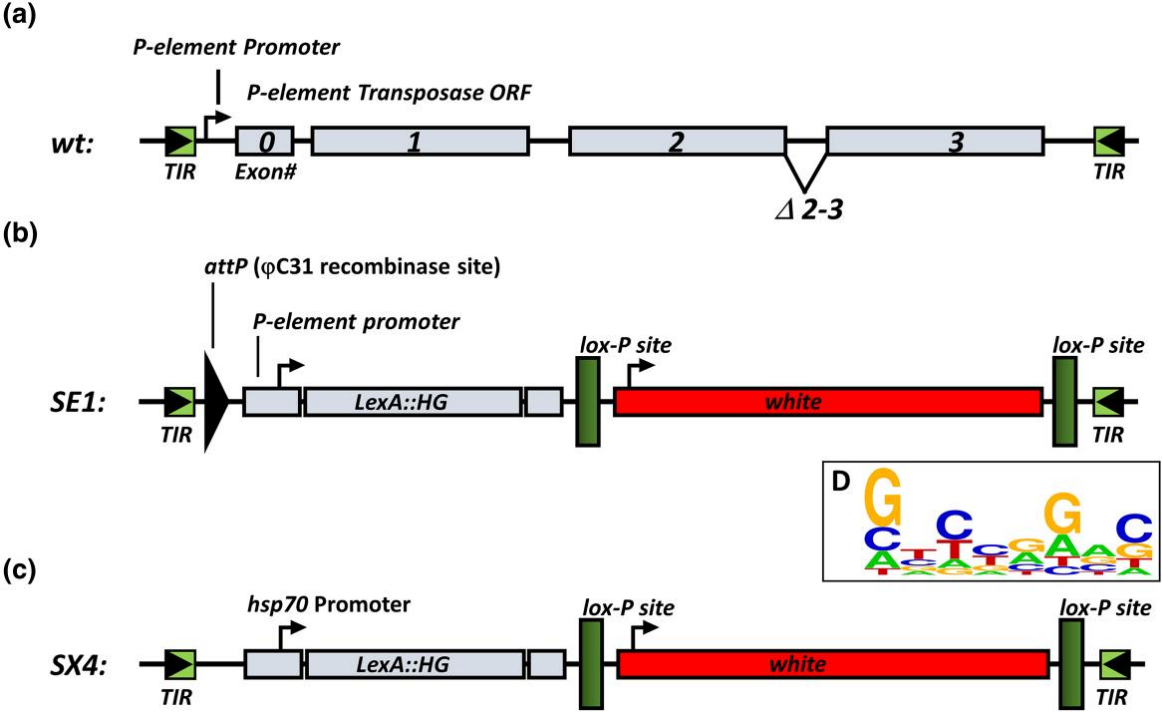
*wt* P-element and lexA enhancer traps. a) *wt* P-element described in O’Hare and Rubin (1983). b) SE1 lexA enhancer trap used in Kockel *et al*. (2019). c) SX4 lexA enhancer trap used in this study. The SX4 enhancer trap encodes a lexA DNA-binding domain fused to the hinge and transactivation domain of Gal4, driven by the hsp70 promoter. The enhancer trap is marked by the *white* selectable eye color marker. See Supplementary Data File 1 for annotated sequence of SX4. d) Sequence logo (see Methods) derived from 281 independent 8 bp direct repeat sequences caused by SX4 insertion.

While prior studies mobilizing the X-linked SE1 element successfully generated LexA enhancer trap flies (Kockel *et al*. 2016, 2019), novel autosomal insertions were recovered at relatively low frequency (<5%) and showed modest expression of LexA. Of note, patterned expression in wing imaginal discs was not recovered, and expression in other tissues was often variegated. To address these limitations, we modified the SE1 element (Methods) to generate the SX4 element (Fig. 1, a and b) and SX4 “starter” fly line. The SX4 P-element carries a LexA::G4 fusion (L = LexA DNA-binding domain; H = Gal4 hinge region; G = Gal4 transcriptional activation domain; together called “LHG”, and referred to as “lexA”) identical to the SE1 P-element (Kockel *et al*. 2016). However, the P-element promoter driving lexA in the SE1 element was replaced by the hsp70 promoter. Thus, compared to the original SE1 transposon (Fig. 1a), the SX4 element has multiple modifications (Supplementary Data File 1 and Fig. 1) including (1) removal of *attB* sequences, (2) replacement of the original P-element promoter with the hsp70 promoter to regulate LexA expression, and (3) placement in the *amnesiac* locus (*amn*) at X:19,887,268, a region with 81 reported, independent transgenic insertions, suggesting permissiveness for P-element transposition.

After transformation of the P{w[mC] = LHGStan-X[SX4]} P-element vector, hereafter referred to as “SX4”, into the *w^1118^* recipient strain (Methods), an index SX4 X-linked transformant was isogenized to the *iso^113232^* genetic background to generate the *w^1118^, SX4; iso#32^II^, iso#32^III^* strain (Methods). Prior studies using the SE1 element in a less-defined genetic background observed insertional bias of the SE1 element to a genomic region containing a KP element, a contaminating nonautonomous P-element derivative with an internal deletion encoding a repressor of transposition (Lee *et al*. 1996, Kockel *et al*. 2019). Thus, we used whole genome sequencing (Methods) with 76× and 80× coverage for males and females (PRJNA912892), respectively, to confirm the absence of KP elements in the *w^1118^, SX4; iso#32^II^; iso#32^III^* strain. Analysis of 8 bp direct repeat sequences from individual SX4 insertions (*n* = 281) shows a slight preference of SX4 towards weak palindromic sites (Fig. 1d), as previously reported (Kockel *et al*. 2019; Linheiro *et al*. 2012).

### Generating novel LexA enhancer-trap lines

To generate LexA-based enhancer trap fly lines, we mobilized the X-linked SX4 P-element to isogenic autosomes *iso#32^II^; iso#32^III^* or the third chromosome SX4 insertion *TM6BSX4^orig^* to the X chromosome (Methods). To facilitate the mobilization of the nonautonomous SX4 P-element, the SX4 donor strain was crossed into a genetic background expressing the activated P-element transposase variant Δ2-3 (Robertson *et al.* 1988). The transposase source was subsequently removed through outcross in the next generation to stabilize the transposition event, generating LexA P-element enhancer-trap lines (Methods; Supplementary Table 1; O’Kane and Gehring 1987). These novel enhancer traps report interactions of its relatively weak hsp70 promoter with the local enhancer environment of the insertion site by LexA expression, displaying spatial and temporal expression specificity (O’Kane and Gehring 1987). We used a similar experimental logic (Methods) to mobilize the SX4 located on III (*TM6B,SX4^orig^*), and isolate LexA insertions in the X-chromosome (Supplementary Table 1).

### Characterization of Stan-X P-element insertion sites

**Fig. 2.**
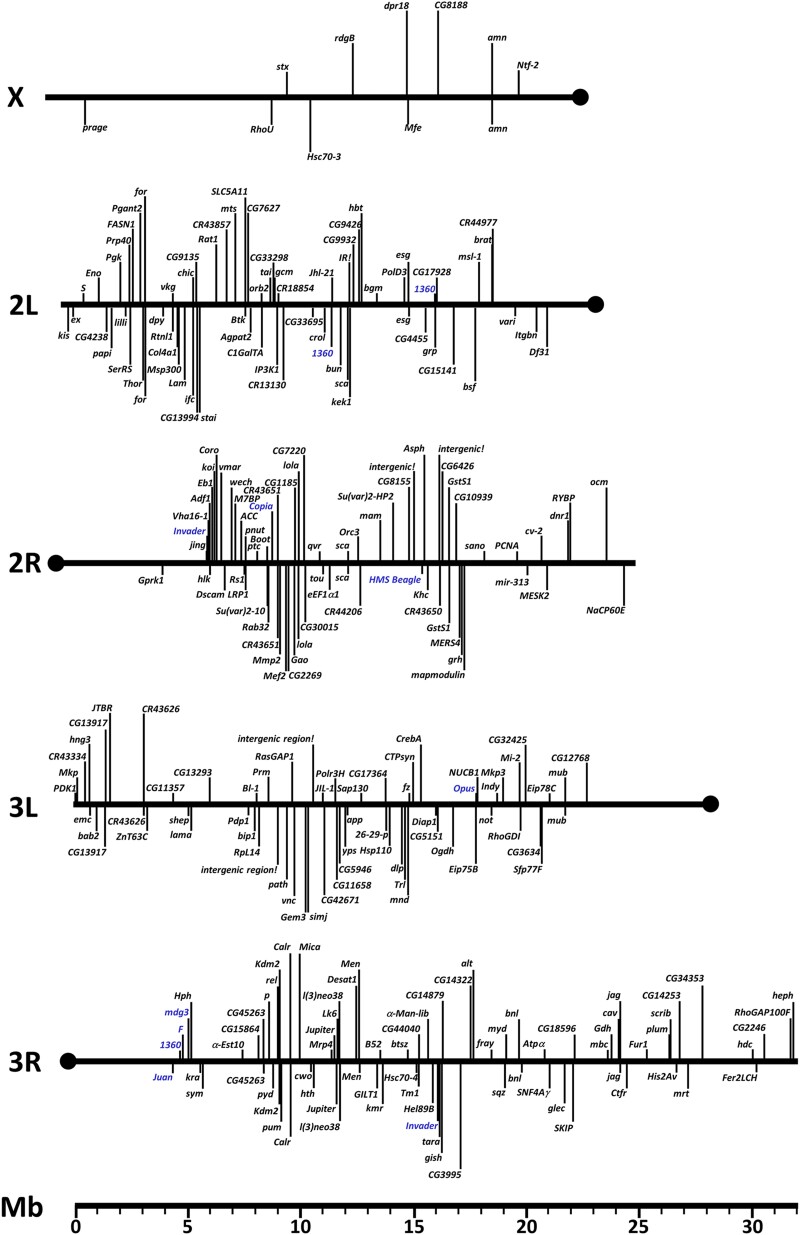
Map of novel Stan-X lexA enhancer trap insertions across chromosomal arms of X, II, and III. Chromosome arms are drawn to scale, and the enhancer trap positions are designated by their molecular coordinates. Scale below is in megabases (Mb). P-element insertions indicated below the chromosomal scaffold are oriented 3′ to 5′, and insertions above are oriented 5′ to 3′ relative to the reference sequence release 6 in FlyBase. Multiple insertions of identical orientation near identical genes are unified as single entry, and are separately listed in Supplementary Table 1. Insertions into natural transposable elements (natural TEs) are indicated in blue.

We next used iPCR-based molecular methods to map the chromosomal insertion position of the Stan-X P-elements to the molecular coordinates of the genomic scaffold (Fig. 2 and Supplementary Table 1). The 301 novel insertions of this study were distributed across autosomes II and III, and their chromosomal arms (2L, 70 insertions; 2R, 69 insertions; 3L, 67 insertions; 3R, 79 insertions). We also isolated 11 insertions on the X-chromosome in a pilot P-element mobilization screen using the *TM6B,SX4^orig^* as a starter line. At 19 loci, multiple P-element insertions (ranging from 2–4) mapped within 1 kb of previously derived lines, supporting the unique value of the individual LexA enhancer traps within the collection. In summary, we identified insertions at 268 unique loci, including one intergenic region (Supplementary Table 1); and all lines were submitted to a fly stock repository (Bloomington, IN).

Natural transposons (natural TEs) constitute a significant portion of repetitive DNA in the genome and represent ∼6% of sequenced euchromatin (Kapitonov and Jurka 2003; Kaminker *et al*. 2002). For the first time, we report 17 out of 301 (5.6%) SX4 lexA enhancer trap insertions into natural transposons, conforming with the expected frequency. This allows ingress to the somatic spatio-temporal expression pattern, if present at all, associated with the repetitive elements at these loci (see below). All tagged natural transposons are present in multiple copies throughout the genome, and represent repetitive DNA. Out of 17 *SX4* enhancer trap insertions into natural TEs, 11 were unambiguously mapped to a specific site within a single copy of a natural TE. Of these 11 natural TEs tagged by *SX4*, seven are present in release6 of the *Drosophila* genome (R6, https://flybase.org/; 1360{}1206, Invader1{}757, Opus{}1033, Juan{}1190, F{}1209, mdg3{}1215, and Invader4{}1371). Four of 11 natural TEs tagged by *SX4* are not represented in R6 of FlyBase and are specific to our *iso^113232^* background (2× 1360, Copia, HMS Beagle, see below, Methods), illuminating differences between the genomes of *iso^113232^* and the FlyBase reference strain *iso^1^*. Four independent SX4 insertions mapped to a cluster of natural TEs (*Juan, F, 1360, mdg3*: Supplementary Table 1) on the pericentromeric-euchromatin boundary at 4.4–5.0 Mbp of chromosomal arm 3R. Six of 17 SX4 insertions into natural TEs could be assigned to a specific clade of natural TEs (insertions into Doc, 2× Opus, 1731, 1360, Rt1a), but could not be unambiguously mapped to a specific location within the *Drosophila* genome due to sequence read limitations during insertion site cloning by iPCR (Supplementary Table 1).

Of the total of 301 novel lexA insertions presented here, the majority (295) were unambiguously mapped by DNA sequencing (295/301, 98%) and integrated into gene elements, including promoters, and the known first exon or intron of transcription units, similar to results from prior work (Bellen *et al*. 2011). Of the 295 mapped insertions presented here, we observed an even distribution of insertional direction by the SX4 P-element into genomic DNA. Using the 5′ and 3′ ends of the SX4 P-element as coordinates, we found that 149/301 insertions were oriented 5′ to 3′, and 146/301 insertions were oriented 3′ to 5′. In six cases, we were unable to determine the direction of P-element insertion. As detailed above, 17/301 (5.6%) insertions of SX4 were in a natural TE (Supplementary Table 1).

The analysis of LexA lines already present within ±1 kb of SX4 insertions sites revealed two loci at which three LexA enhancer traps were previously generated; *escargot* (*esg*) and *SNF4γ*, which encodes the AMPK subunit gamma. These loci are known hotspots for P-element insertion, and the current study identifies three additional independent insertions into *esg*, all in the promoter region of that gene. Three SX4 insertions integrated in loci previously tagged twice with LexA insertions (*CG33298*, *α-Est10, lncRNA:CR43626*), and thirty-seven SX4 insertions of this study mapped within ±1 kb of the insertion site for one prior LexA enhancer trap (Supplementary Table 1). In summary, our approach generated multiple novel LexA-based autosome and sex chromosome enhancer traps.

### Tissue expression patterns of LexA from SX4 insertions

**Fig. 3.**
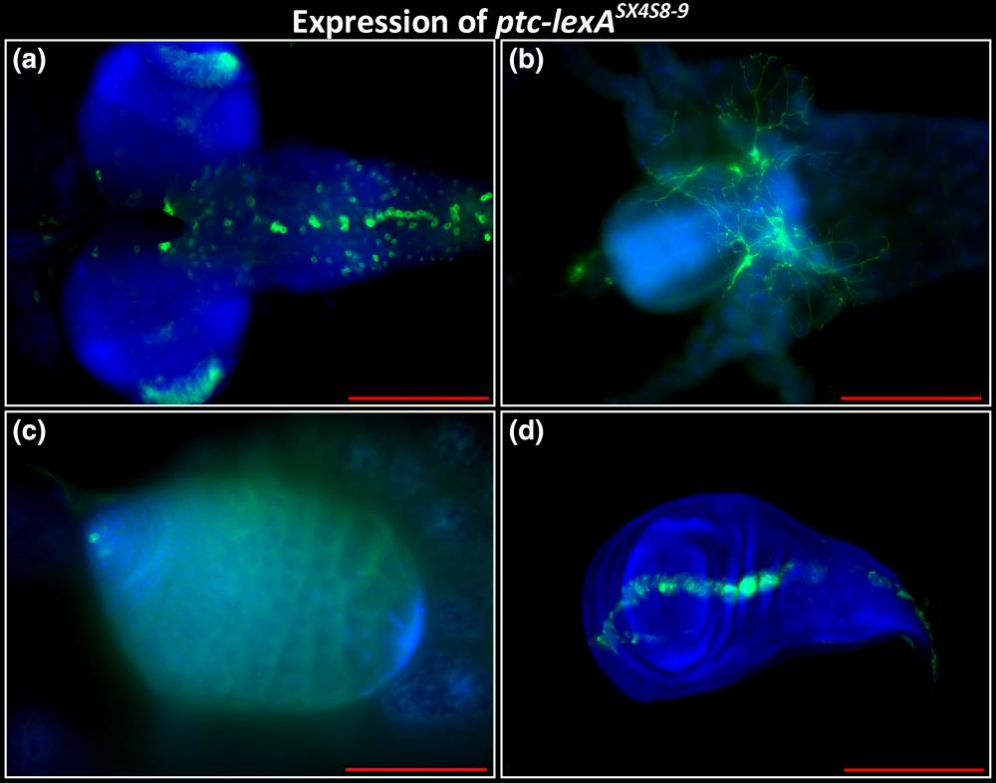
Expression of SX4 enhancer trap insertion into *patched*, *ptc-lexA^SX4S8–9^.* Genotype: *ptc-lexA^SX4S8–9^/+; lexAop-CD8::GFP*. a) Third instar larval brain, expression in VNC and CNS. b) Third instar larval gut, expression in enteric neurons located at proventriculus, caeca, and midgut. c) Third instar larval testis, expression in putative hub cells and spermatocytes. d) Third larval instar wing disc. Expression along the putative anterior–posterior boundary. Anterior to the right, except d) ventral to the right. Blue: DAPI. Green: anti-GFP. Scale bar 200 μm, except c) 100 μm.

**Fig. 4.**
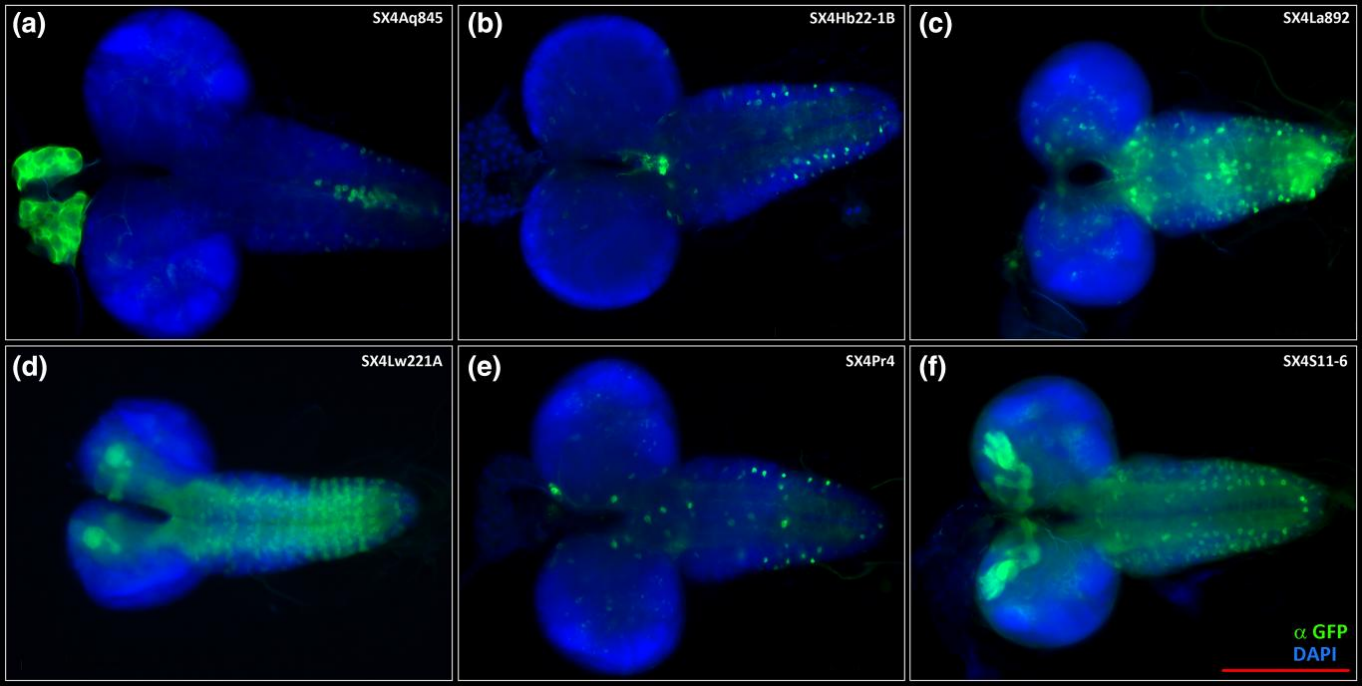
Expression pattern of 6 representative SX4 enhancer traps crossed to lexAop-CD8::GFP in wandering third instar larval brains by IHC. Green: anti-GFP, blue: DAPI. a) *SX4Aq845; lexAop-CD4::GFP*. b) *SX4Hb22-1B; lexAop-CD4::GFP*. c) *SX4La892; lexAop-CD4::GFP*. d) *SX4Lw221A; lexAop-CD4::GFP*. e) *SX4Pr4; lexAop-CD4::GFP*. f) *SX4S11-6; lexAop-CD4::GFP*. All images were recorded with a 20× lens, scale bar = 200 μm.

**Fig. 5.**
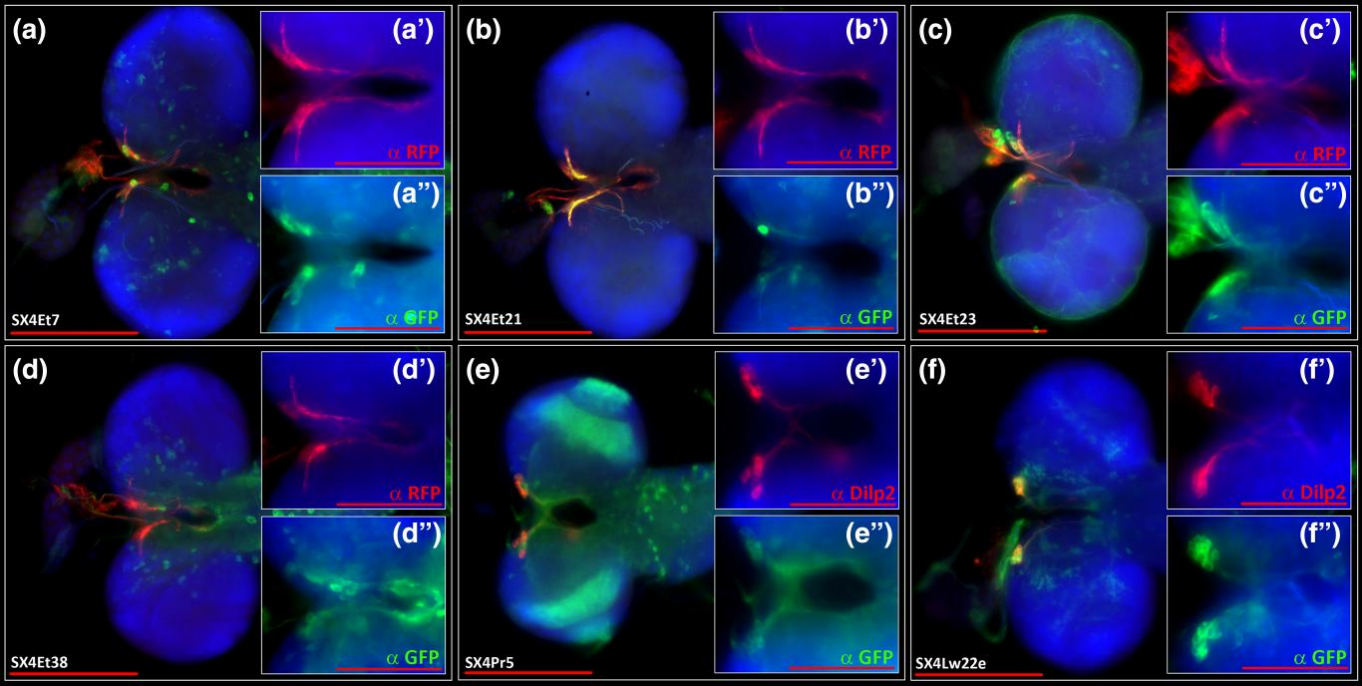
Immunohistochemistry analysis of lex-A activity in insulin expressing cells (IPCs) of selected SX4 enhancer-trap lines. IPCs are marked by dilp2-Gal4, UAS-CD4::tdt (a–d), or anti-dilp2 co-stain (e and f), shown in red. Main images (a–f) were recorded with a 20× lens, scale bar = 200 μm. Inserts were recorded with 40× lens (A’, A”–F’, F”), scale bar = 100 μm. Green: anti-GFP. Red: anti-RFP (a–d) or anti-ilp2 (e and f). Blue: DAPI. A, A’, A”) *dilp2-Gal4, UAS-CD4:tdt; SX4Et7; lexAop-CD8::GFP*. B, B’, B”) *dilp2-Gal4, UAS-CD4:tdt; SX4Et21; lexAop-CD8::GFP*. C, C’, C”) *dilp2-Gal4, UAS-CD4:tdt; SX4Et23; lexAop-CD8::GFP*. D, D’, D”) *dilp2-Gal4, UAS-CD4:tdt; SX4Et38; lexAop-CD8::GFP*. E, E’, E”) *SX4Pr5; lexAop-CD8::GFP*. F, F’, F”) *SX4Lw22e; lexAop-CD8::GFP*.

Larval growth from stage 1 (L1) to stage 3 (L3) is facilitated by intermittent exoskeletal shedding, resulting in wandering third instar larvae prior to pupariation. To verify enhancer trapping by the SX4 P-element, we intercrossed novel insertion lines with flies harboring a “reporter” transgene encoding LexAop linked to a cDNA encoding a membrane-GFP (LexAop2-CD8::GFP; Pfeiffer *et al*. 2010), then confirmed membrane-associated GFP expression in tissues dissected from L3 larvae (Methods, Figs. 3–5, Supplementary Table 1, Kockel *et al*. 2016). We analyzed wandering 3rd instar larvae of bi-transgenic offspring (hereafter referred to as SX4>lexAop-GFP) after immunohistochemistry (IHC) staining for GFP, and simultaneous counter-staining for cell nuclei (DAPI). Images from selected SX4>lexAop-GFP tissues were collected, and tissue expression catalogued (Supplementary Table 1). Within the collection, we detected GFP expression in multiple tissues of L3 larva, including neuronal cell types in the central nervous system (CNS), ventral nerve cord (VNC), and peripheral nervous system, imaginal discs, and a wide range of other somatic tissues like fat body, Malpighian tubules, trachea, and ring gland (Supplementary Table 1).

*patched* (*ptc*) has a well-studied transcriptional expression pattern (Phillips *et al*. 1990, Supplementary Table 1, Fig. 3), and *ptc-Gal4* is among the most-referenced Gal4 lines in the *Drosophila* literature (https://flybase.org/GAL4/freq_used_drivers/). To test if a lexA enhancer trap in *ptc* reproduced the known expression pattern, we analyzed line *SX4S8-9*, located near the transcriptional start of *ptc* (*ptc-lexA^SX4S8–9^)* in 2L at 44D1. Analysis of third instar wing discs revealed a LexA expression domain from *ptc-lexA^SX4S8–9^* along the anterior–posterior boundary, as described for *ptc* RNA whole mount in situ hybridization of wing imaginal discs (Phillips *et al*. 1990). In addition, we observed expression in the CNS and VNC, enteric neurons, and putative hub cells and spermatocytes of the larval testis, consistent with prior reports (Fig. 3, Li *et al*. 2022). In summary, LexA expression from the *SX4S8-9* enhancer-trap element inserted into the *ptc* locus reproduced several features of *ptc* expression across diverse somatic and germ line tissues and cell types.

To address if distinct lexA enhancer trap insertions produce distinct expression patterns, we surveyed expression of LexA by imaging the membrane-tagged GFP reporter in L3 larval brains and associated tissues like the ring gland of 6 independent SX4 insertions. For example, *LexAop-CD8::GFP* expression was directed by LexA from an insertion in *CG9426* (*SX4Aq854*), *bsf/Ntf-2r* (*SX4Hb22-1*), *lola* (*Sx4Lw221A*), and *vnc* (*SX4Pr4*). We observed distinct patterns of cell labeling in the CNS, VNC, and ring gland (Fig. 4, a–f), in accordance with SX4 enhancer trap insertions located in distinct loci of the *Drosophila* genome give rise to distinct expression patterns.

To facilitate accessibility of all molecular and imaging data, including supplementary images (Supplementary Table 1), we uploaded these to a database (https://stanx.stanford.edu), searchable by expression pattern, cytology, and specific genes.

### Identification of SX4 lines that express LexA in insulin-secreting neurons

Systemic insulin in *Drosophila* emanates from a paired cluster of neurons in the *pars intercerebralis* comprised of 12–14 insulin-producing cells (IPCs: Fig. 5, a–f). IPCs express genes encoding *Drosophila* insulin-like peptides (Ilp’s), including *ilp-2, ilp-3*, and *ilp-5* (Brogiolo *et al*. 2001; Rulifson *et al*. 2002; Li *et al*. 2022). Prior enhancer trap studies identified homogeneous LexA expression in these insulin^+^ cells, suggesting shared regulatory features within individual IPCs. However, we noted heterogeneous expression of the SX4Et7 enhancer trap, with expression of the LexAop::GFP reporter only in a subset of 1–2 IPCs (green, Fig. 5a”) within the cluster of Ilp2^+^ IPCs (red, Fig. 5a’). To investigate the possibility of heterogeneous genetic regulation in IPCs, we screened 87 lines and identified 16 additional lines with LexA activity in IPCs, identified by expression of the LexAop::GFP reporter in Ilp2^+^ IPCs (Fig. 5 and Supplementary Table 1).

To facilitate localization of LexA activity in IPCs, we co-labeled IPCs using antibody to Ilp-2 or by specific marking of IPCs with *ilp2-Gal4* driving *UAS-CD4::tdTomato*. Of note, we observed insertions that express LexAop-CD8::GFP throughout the entire IPC cluster (e.g. Figure 5f”, SX4Lw22e, *insertion in mayday*; Supplementary Table 1), and insertions that express LexAop-CD8::GFP in a subset of IPCs only (e.g. Figure 5b”, SX4Et2, insertion in *kis*).

We selected six genes trapped by an SX4 insertion (Fig. 5: SX4Et7 in *B52*; SX4Et21 in *kis*; SX4Et23 in *Afd1*; SX4Et38 in *Hel89B*; SX4Pr5 in *Star*; and SX4Lw22e in *myd*) that were confirmed to drive expression in IPCs and cross-referenced expression of that gene using IPC gene expression data from the Fly Cell Atlas (FCA, Li *et al*. 2022). The IPC FCA data is based on single-nuclei RNAseq (snRNAseq) of fluorescence activated cell sorting-sorted IPC nuclei labeled by *ilp2-Gal4; UAS-2xunc84::GFP* (Li *et al*. 2022). Single-nuclei libraries from IPCs had robust expression of *ilp-2* confirming their IPC identity (Supplementary Fig. 2). In total, snRNAseq data from 473 IPC nuclei (232 male, 241 female) were correlated to the IPC expression of genes tagged by the selected enhancer traps, including all six cases detailed in Fig. 5. For example, *kis* was expressed in 362/473 nuclei, while *Adf1* was expressed in 41/473 nuclei. In summary, snRNAseq data of IPCs confirms the expression of genes tagged by SX4 LexA enhancer traps in IPCs.

### Evidence of RNA and *LexA* expression from natural TEs in somatic cells

**Fig. 6.**
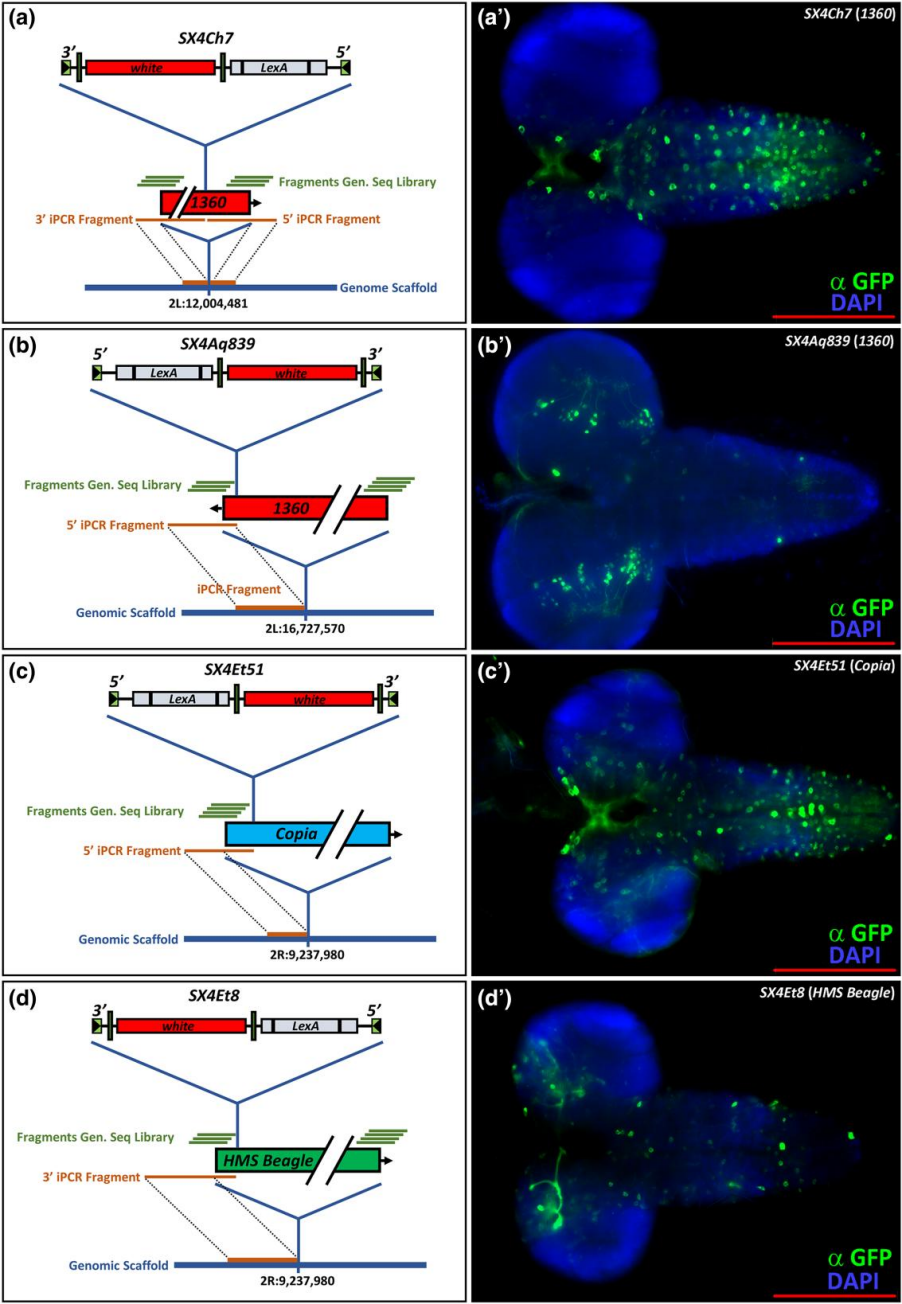
Location, tagging, breakpoint cloning, and L3 brain expression pattern of natural TEs not present in FlyBase R6. a–d) Schematic representation of natural TE locations not represented in R6 and associated data. a) *1360* located at 2L:12,004,481 tagged by SX4Ch7, integrated 501 bp off the 3′ end of 1360. b) *1360* located at 2L:16,727,570 tagged by SX4Aq839, integrated 50 bp 3′ off the 3′ end of *1360*, c) *Copia* located at 2R:9,237,980 tagged by SX4Et51, integrated 174 bp into 5′ of *Copia*, d) *HMS Beagle* located at 2R:15,951,007 tagged by SX4Et8 integrated 133 bp off the 5′ end of *HMS Beagle*. Blue line: genomic scaffold. Orange line: sequence originated by iPCR, spanning the breakpoint of the SX4 enhancer trap and the natural TE, and the breakpoint of the natural TE with the genomic scaffold. iPCR representation not to scale. Green lines: sequenced amplicons from *w^1118^, SX4; iso#32^II^; iso#32^III^* genomic libraries, found by TE mapper (Methods). Sequence of amplicons is listed in Supplementary Table 2. Amplicon representations not to scale. Red box: natural TE. Arrow: direction of natural TE 5′–3′. A’–D’) Third instar larval brains of respective LexA enhancer traps crossed to *w; lexAop-CD8::*GFP. Genotypes: A’) *w; SX4Ch7/+; lexApo-CD8GFP/+*. B’) *w; SX4Aq839/+; lexApo-CD8GFP/+*. C’) *w; SX4Et51/+; lexApo-CD8GFP/+*. D’) *w; SX4Et8/+; lexApo-CD8GFP/+*. Blue: DAPI, Green: anti-GFP. Scale bar: 200 μm.

Robust repression of natural TE sequences in both somatic and germ line cells has been reported (Czech *et al*. 2018; van den Beek *et al*. 2018). We therefore addressed if somatic expression of lexA derived from SX4 enhancer traps integrated into natural TEs was detectable. We analyzed the L3 brain expression patterns of four independent lines harboring a SX4 element integrated into distinct natural TEs: two different *1360* elements (tapped by *SX4Ch7* and *SX4Aq839*, respectively), *Copia* (*SX4Et51*), and *HMS Beagle* (*SX4Et8*) (Fig. 6, Supplementary Tables 1 and 2; Methods). Analysis of *LexAop-CD8::GFP* expression driven by these lines revealed a clearly detectable presence of lexA activity in L3 brains, as well as distinct expression patterns of the SX4 lines from each other (Fig. 6). This includes *SX4Et8*, which was expressed in IPCs (Fig. 6d, see below), and *SX4Ch7* and *SX4Aq839,* independent insertions into 1360 elements that are positioned at different genomic locations (Fig. 6, a and b). These findings suggest that the genomic location, rather than the TE itself, influences LexA expression of the SX4 insertion (Treiber and Waddell 2020).

To independently confirm somatic expression of natural TEs, we analyzed snRNAseq data from IPCs and CC cells for amplicons aligning to natural TEs present in the *Drosophila* genome (Methods, Treiber and Waddell 2020, Li *et al*. 2022). This analysis revealed RNAseq amplicons aligning to natural TEs in snRNAseq libraries derived from IPCs and CC cells. On average, ∼5% and ∼2% of the overall RNA content of CC cells and IPC snRNAseq libraries, respectively, map to natural TE sequences (Supplementary Table 1 and Supplementary Fig. 2). We stratified this expression to two “classes” of natural TE: class II DNA cut-and-paste TEs (*1360*) and class I retrotransposons (*Invader1, Opus, Juan, F, mdg3*, and *Invader4*; Supplementary Fig. 2) and found both classes of natural TEs expressed in CCs and IPCs. The presence of multiple TE copies precluded unambiguous determination of expression levels derived from a single specific natural TE insertion. In summary, the RNA expression from natural TEs as determined by snRNAseq suggests somatic RNA expression derived from natural TEs in CC cells and IPCs (Tsurumi and Li 2019, Copley and Shorter 2023, Lindehell *et al*. 2023). The specific expression patterns in L3 brains of SX4 enhancer traps inserted into natural TEs support the hypothesis of a local mechanism of derepression in this early life stage.

### An international scholastic network to generate resources for *Drosophila* genetics

**Fig. 7.**
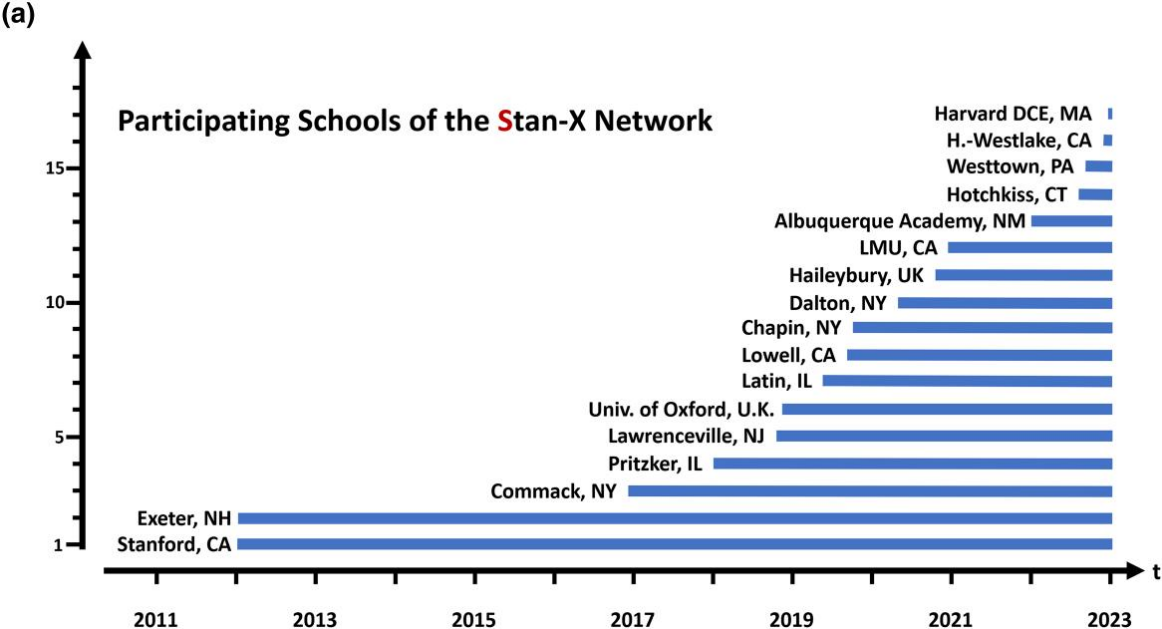
The Stan-X program. a) Timeline of school recruitment into the Stan-X program. Recruitment takes place during the school year, followed by training of the teachers in the Discover Now Teacher Academy in the summer (Supplementary Fig. 1). See text for details.

In our prior studies, we produced and characterized novel fly enhancer-trap lines through an interscholastic partnership of secondary school and university-based researchers in the United States (Kockel *et al*. 2016, 2019; Chang *et al*. 2022). This involved development of curricula permitting flexible scheduling of three laboratory-based “modules”. These are comprised of fly intercrosses and P-element mobilization (module 1), molecular biology and enhancer-trap mapping (module 2), and immunofluorescence-based microscopy of dissected larval tissues to confirm LexA activity in specific tissues (module 3: Methods). In some schools, this occurred in year-long courses (Supplementary Fig. 1A), while in other schools, specific elements like mapping of P-element genomic insertions, or genome sequencing, were achieved by shorter classes (Kockel *et al*. 2019; Methods). This curricular model integrated and enhanced the longitudinal quality of genetic experiments performed across years at specific schools (flies generated in one course could be characterized the following semester by another set of students). This prior work also demonstrated effective, productive collaborations of students and instructors across institutions (for example, flies generated in one school could be shared with another school that performed molecular mapping studies). Over a 10-year span (2012–2022), this network of collaborating schools has expanded to 17 schools (Fig. 7).

To meet growth of the Stan-X network and demand for teacher training, the Teacher Academy “Discovery Now” was instituted in 2018. Incoming teachers receive a 2-week intensive training, one week online, one week in person (Supplementary Fig. 1B). This course prepares new instructors to implement the Stan-X research curriculum of molecular biology and genetics, and provides grounding in essential course logistics like equipment acquisition. This summer training for instructors is provided annually (www.Stan-X.org).

Here, we assessed if the curriculum of *Drosophila-based* genetics, molecular biology, genomics, and tissue analysis framing original, high-quality research could be adopted at additional secondary schools and universities, including abroad. As indicated by the data and resources detailed here, our studies show that research at secondary schools and universities in the United States and United Kingdom fostered production and sharing of data and fly strains, and achievement of student learning goals.

In this study, two hundred ninety-four high school students, thirty-two high school teachers, and staff supervised Stan-X classes at fourteen secondary schools. Additional students and instructors participated in Stan-X classes at three universities. Most SX4 enhancer-trap lines were generated during the fall to spring terms in school years 2019–2022. All participating schools executed insertion site cloning and, equipment allowing, characterized tissue expression patterns. Ten high school students finished the characterization of the enhancer trap collection during summer internships in the Kim laboratory at Stanford University.

In addition to curricular development at these schools, these interscholastic partners benefitted from structured interactions with network leaders at Stanford, Lawrenceville, and Exeter that included weekly research teleconferences with course instructors and classes during the school year. In addition, there were university-based summer internships for students (*n* = 21) or instructors (*n* = 6), and development of annual student-led conferences with participants from multiple schools for presenting data and curricular innovations. In turn, university collaborators made regular visits to secondary school classes during the school year (Methods).

There were also multiple positive outcomes for students and teachers at partnering schools that were unanticipated. These included (1) emergence of *student* course alumni as *instructors* at their home institution or another Stan-X partner site (*n* = 6), (2) interscholastic collaboration and data development through sharing of Stan-X fly strains and other resources, (3) regular video conferencing and in-person multi-institutional student symposia organized independently by Stan-X instructors, (4) additional professional development opportunities for adult teachers, including presentation of pedagogy at professional meetings, promotion, and travel to other Stan-X partner schools, (5) development of new courses founded on Clustered Regularly Interspaced Short Palindromic Repeats and other approaches to generate novel LexA or LexAop strains (Chang *et al*. 2022; Wendler *et al*. 2022) or fly genomics (see Kockel *et al*. 2019), (6) development of Stan-X summer school courses at Harvard, Oxford, Lawrenceville, and Exeter (Supplementary Fig. 1B, https://stan-x.org), and (7) philanthropic funding for science curricular innovation and infrastructure modification to Stan-X partners. Thus, an intercontinental consortium of students and instructors at secondary schools and university-based programs have formed a unique research network actively generating novel fly strains suitable for investigations by the community of science.

## Discussion

Here, we introduced a novel lex-A enhancer trap construct in a unique isogenic background (*iso^113232^*) and used this element to generate more than 300 novel LexA enhancer trap insertions through scholastic courses at seventeen institutions. We characterized gene expression of a substantial fraction of these insertions in 3rd instar larval organs or tissues like, CNS, VNC, and gut, with a special emphasis on IPC expression in the larval CNS. Future studies could address similarities and differences of independent derived enhancer-trap lines with a similar insertion site (e.g. a region of 1 kb). We also generated a SX4 P-element on the third chromosome *TM6B* balancer (*TM6B, SX4^orig^*) that was successfully mobilized for selection of X-linked enhancer traps. Analysis of the SX4 insertion site sequence, insertion directions, and genomic insertion sites associated with the 5′ end of transcription units revealed a similar profile compared to the first-generation SE1 lexA enhancer trap (Kockel *et al*. 2019). Thus, the new SX4 LexA enhancer-trap element has multiple hallmarks of a P-element insertion vector. In summary, the new SX4 “starter” lines represent an advance over the prior SE1 LexA enhancer-trap line (Kockel *et al*. 2016).

Prior work revealed the presence of KP elements as an impediment for P-element mobilization experiments (Kockel *et al.* 2019) reflecting: (1) bias for replacement of the KP element by the enhancer trap P-element, (2) KP mobilization by Δ2-3 transposase giving rise to uncontrolled genetic heterogeneity, and (3) dominant-negative effect of KP on Δ2-3 transposase, lowering the overall transposition rate per male germ line. Here, we used the genetic background *iso^113232^*, where we confirmed the absence of an autosomal KP element by genomic sequencing. In this genetic background, as predicted, we observed increased transposition frequency and more randomized insertional distribution of our SX4 starter P-element across the chromosomes. These features have also improved workflows in participating school courses.

Each natural transposon family is present in multiple identical, or nearly-identical copies of DNA sequence per genome; thus, unambiguous mapping of insertions within these repetitive sequences requires special strategies. Here, we report the index, successful mapping of SX4 P-element insertions into natural transposons. We successfully mapped 11/17 SX4 insertions in natural TE sequences, as well as the position of the SX4-tagged natural TE within the *Drosophila* genome. Of note, 4/11 natural TEs that were tagged by SX4 and positionally identified are not represented in the current release 6 of the *Drosophila* genome. Therefore, these represent unique natural TEs copies specific to the *iso^113232^* background.

Intact natural TEs encode transposase enzyme (class II, cut-and-paste TEs), or other factors mediating replication and insertion (class I, RNA transposons; McCullers and Steiniger 2017). To prevent mobilization and genome instability, the transcription of natural TEs is thought to be repressed in somatic and germ line tissues (Senti and Brennecke 2010; Czech *et al*. 2018; van den Beek *et al*. 2018). However, SX4 enhancer traps tagging natural TEs showed clear *LexA* expression in a variety of somatic cells, including L3 neurons, indicating the accessibility of transcriptional machinery to the genomic locus harboring the TE. This is consistent with observations of active transcription of natural TEs in the *Drosophila* adult brain (Treiber and Waddell 2020, Lindehell *et al*. 2023), increased natural TE expression in aged flies (Tsurumi and Li 2020, Yang *et al*. 2022), and our analysis of TE expression by snRNAseq in IPCs and CC cells. This suggests that somatic transcription and, possibly, transposition of natural TEs might occur in somatic tissue in vivo (Siudeja *et al*. 2021; Yang *et al*. 2022, Copley and Shorter 2023).

Experimental biology benefits from temporal- or cell type-specific control of gene expression, exemplified by the binary expression strategies pioneered in the *Drosophila* GAL4-UAS system (Brand and Perrimon 1993). Intersectional approaches, like simultaneous use of the LexA-LexAop and GAL4-UAS systems, have also greatly enhanced experimental and interpretive power in fly biology, particularly studies of neuroscience and intercellular communication (Simpson 2016; Dolan *et al*. 2017; Martín and Alcorta 2017). Thus, new LexA enhancer-trap lines presented here significantly expand the arsenal of available LexA expression tools (Pfeiffer *et al*. 2010; Kockel *et al*. 2016). Prior studies have demonstrated that P-element insertion in flies is nonrandom (O’Hare and Rubin 1983; Berg and Spradling 1991; Bellen *et al*. 2011), with a strong bias for transposition to the 5′ end of genes (Spradling *et al*. 1995). Here and in prior work, we have found a similar preference with SX4 P-element transposition; 89% of unique insertions were located in the promoter or 5′ UTR regions of genes. Molecular characterization and studies of LexAop-regulated GFP reporter genes indicate that the enhancer traps described here are distinct, with LexA expressed in multiple tissues, including the CNS, VNC, fat body, and muscle. These enhancer-trap lines were submitted to the Bloomington Stock Center to enhance resource sharing.

The resources and outcomes described here significantly extend and develop the interscholastic partnership in experiment-based science pedagogy described in our prior studies (Kockel *et al*. 2016, 2019), which previously involved Stanford University researchers and biology classes at four US secondary schools. A scholastic network, called Stan-X, now links university researchers with secondary school and undergraduate students and teachers around the world. The Stan-X network used P-element mobilization in *Drosophila melanogaster* to generate LexA enhancer-trap lines reported here. Curricula based on fruit fly genetics, developmental and cell biology, and molecular biology provided a practical framework for offering authentic research experiences for new scientists detailed previously (Kockel *et al*. 2016, 2019; Redfield 2012). Important research and educational goals, including a keen sense of “ownership” of problems (Hatfull *et al*. 2006) and discovery, were achieved because the outcomes from experiments were “unscripted”. In addition, work permitted students and instructors to create tangible connections of their experimental outcomes (data, new fly strains) to a global science community. Data and tools from this international scholastic network demonstrated how university research laboratories can collaborate with community partners, including with resource-challenged schools serving youth under-represented in science, to innovate experiment-based STEM curricula and experiential learning that permit discovery, the *sine qua non* of science.

Indices of practical outcomes from our work include steady requests for LexA enhancer-trap lines (currently >450 Stan-X lines) from the Drosophila Bloomington Stock Center, and Stan-X fly strain use has been cited in 24 publications since 2016 (e.g. Lee *et al*. 1996; Cohen *et al*. 2018; Zhou *et al*. 2020; Ribeiro *et al*. 2022). Our interscholastic partnerships and classroom-based research have expanded to include high schools and universities on multiple continents (Fig. 7). The secondary schools encompass a spectrum of public, charter, independent and “high needs” schools, with day or boarding students. Three Stan-X partners are in public high schools serving ethnically and economically diverse urban communities (Lowell, San Francisco; Pritzker, Chicago; and Commack, Long Island, NY), while the remainder are independent secondary schools or private universities. This experience demonstrates the feasibility and challenges of expanding the Stan-X model to public schools, which have unique resource challenges. Stan-X programs have instructed seven hundred fifty-two students since 2012, 67% female. At independent schools, 55% of Stan-X students were female (*n* = 562); at public high schools, 70% were female (*n* = 190). These findings suggest that curriculum-based experimental science programs like Stan-X could help address persistent gender-based disparities in science, though this possibility requires further study with case controls. Similar to the experience of others (DrosAfrica 2020), we have found that the Stan-X curriculum can also be used abroad to foster *Drosophila-*based pedagogy. Additional work outside the scope of this study is also needed to assess the longitudinal impact of programs like Stan-X on ethnic or socio-economic disparities in the scientific workforce.

In summary, this experience demonstrates the feasibility of developing productive global partnerships between schools to foster experience-based science instruction with a powerful experimental genetic organism. The thriving partnerships described here form a dynamic network of instructors, students, classes, and school leaders that have produced useful science, and enhanced the personal and professional growth and development of its participants.

## Supplementary Material

jkad124_Supplementary_Data

## Data Availability

All Stan-X SX4 derivatives and associated data are available at the Bloomington Stock Center. The genome sequence data of w[1118], SX4; iso#32[II]; iso#32[III] is available on SRA https://www.ncbi.nlm.nih.gov/sra/PRJNA912892, or accession number PRJNA912892. All molecular and image data are additionally available at https://stanx.stanford.edu. Course manuals, scaffolding problem sets, and sample course daily and weekly schedules are available on request. Supplemental material available at G3 online.
