# Supplementary material for: Generation of LexA enhancer-trap lines in Drosophila by an international scholastic network
Source: G3 (Bethesda). 2023 Jun 6;13(9):jkad124. doi: 10.1093/g3journal/jkad124 (PMC10468311; doi:10.1093/g3journal/jkad124)
Supplement: jkad124_Supplementary_Data [file jkad124_supplementary_data.zip › Data_File_S1_G3-2023-404154.pdf]

1 CCGCTGGACT ACGTGGGTCT GGCCCATGAT GAAATAACAT AAGGTGGTCC CGTCGATAGC

61 CGAAGCTTAC CGAAGTATAC ACTTAAATTC AGTGCACGTT TGCTTGTTGA GAGGAAAGGT

121 TGTGTGCGGA CAATTTTTTT TTGAAAACAT TAACCCTTAC GTGGAATAAA AAAAAATGAA

181 ATATTGCAAA TTTTGCTGCA AAGCTGTGAC TGGAGTAAAA TTAATTCACG TGCCGAAGTG

241 TGCTATTAAG AGAAAATTGT GGGAGCAGAG CCTTGGGTGC AGCCTTGGTG AAAACTCCCA

301 AATTTGTGAT ACCCACTTTA ATGATTCGCA GTGGAAGGCT GCACCTGCAA AAGGTCAGAC

361 ATTTAAAAGG AGGCGACTCA ACGCAGATGC CGTACCTAGT AAAGTGATAG AGCCTGAACC

421 AGAAAAGATA AAAGAAGGCT ATACCAGTGG GAGTACACAA ACAGAGTAAG TTTGAATAGT

481 AAAAAAAATC ATTTATGTAA ACAATAACGT GACTGTGCGT TAGGTCCTGT TCATTGTTTA

541 ATGAAAATAA GAGCTTGAGG GAAAAAATTC GTACTTTGGA GTACGAAATG CGTCGTTTAG

601 AGCAGCAGCC GAATTCGGTA CCGAGCGCCG GAGTATAAAT AGAGGCGCTT CGTCTACGGA

661 GCGACAATTC AATTCAAACA AGCAAAGTGA ACACGTCGCT AAGCGAAAGC TAAGCAAATA

721 AACAAGCGCA GCTGAACAAG CTAAACAATC TGCAGTAAAG TGCAAGTTAA AGTGAATCAA

781 TTAAAAGTAA CCAGCAACCA AGTAAATCAA CTGCAACTAC TGAAATCTGC CAAGAAGTAA

841 TTATTGAATA CAAGAAGAGA ACTCTGAATA GATCTGCGGC CGCTAAGCAA ATAAACAAGC

901 GCAGCTGAAC AAGCTAAACA ATCTGCAGCC CAAGCTTGAA GCAAGCCTCC TGAAAGATGA

961 AAGCGTTAAC GGCCAGGCAA CAAGAGGTGT TTGATCTCAT CCGTGATCAC ATCAGCCAGA

1021 CAGGTATGCC GCCGACGCGT GCGGAAATCG CGCAGCGTTT GGGGTTCCGT TCCCCAAACG

1081 CGGCTGAAGA ACATCTGAAG GCGCTGGCAC GCAAAGGCGT TATTGAAATT GTTTCCGGCG

1141 CATCACGCGG GATTCGTCTG TTGCAGGAAG AGGAAGAAGG GTTGCCGCTG GTAGGTCGTG

1201 TGGCTGCCGG TGAACCACTT CTGGCGCAAC AGCATATTGA AGGTCATTAT CAGGTCGATC

1261 CTTCCTTATT CAAGCCGAAT GCTGATTTCC TGCTGCGCGT CAGCGGGATG TCGATGAAAG

1321 ATATCGGCAT TATGGATGGT GACTTGCTGG CAGTGCATAA AACTCAGGAT GTACGTAACG

1381 GTCAGGTCGT TGTCGCACGT ATTGATGACG AGGTTACCGT TAAGCGCCTG AAAAAACAGG

1441 GCAATAAAGT CGAACTGTTG CCAGAAAATA GCGAGTTTAA ACCAATTGTC GTAGATCTTC

1501 GTCAGCAGAG CTTCACCATT GAAGGGCTGG CGGTTGGGGT TATTCGCAAC GGCGACTGGC

1561 TGGGATCCCC CGTACAAGAT AATGTGAATA AAGATGCCGT CACAGATAGA TTGGCTTCAG

1621 TGGAGACTGA TATGCCTCTA ACATTGAGAC AGCATAGAAT AAGTGCGACA TCATCATCGG

1681 AAGAGAGTAG TAACAAAGGT CAAAGACAGT TGACTGTATC GATTGACTCG GCAGCTCATC

1741 ATGATAACTC CACAATTCCG TTGGATTTTA TGCCCAGGGA TGCTCTTCAT GGATTTGATT

1801 GGTCTGAAGA GGATGACATG TCGGATGGCT TGCCCTTCCT GAAAACGGAC CCCAACAATA

1861 ATGGGTTCTT TGGCGACGGT TCTCTCTTAT GTATTCTTCG ATCTATTGGC TTTAAACCGG

1921 AAAATTACAC GAACTCTAAC GTTAACAGGC TCCCGACCAT GATTACGGAT AGATACACGT

1981 TGGCTTCTAG ATCCACAACA TCCCGTTTAC TTCAAAGTTA TCTCAATAAT TTTCACCCCT

2041 ACTGCCCTAT CGTGCACTCA CCGACGCTAA TGATGTTGTA TAATAACCAG ATTGAAATCG

2101 CGTCGAAGGA TCAATGGCAA ATCCTTTTTA ACTGCATATT AGCCATTGGA GCCTGGTGTA

2161 TAGAGGGGGA ATCTACTGAT ATAGATGTTT TTTACTATCA AAATGCTAAA TCTCATTTGA

2221 CGAGCAAGGT CTTCGAGTCA GGTTCCATAA TTTTGGTGAC AGCCCTACAT CTTCTGTCGC

2281 GATATACACA GTGGAGGCAG AAAACAAATA CTAGCTATAA TTTTCACAGC TTTTCCATAA

2341 GAATGGCCAT ATCATTGGGC TTGAATAGGG ACCTCCCCTC GTCCTTCAGT GATAGCAGCA

2401 TTCTGGAACA AAGACGCCGA ATTTGGTGGT CTGTCTACTC TTGGGAGATC CAATTGTCCC

2461 TGCTTTATGG TCGATCCATC CAGCTTTCTC AGAATACAAT CTCCTTCCCT TCTTCTGTCG

2521 ACGATGTGCA GCGTACCACA ACAGGTCCCA CCATATATCA TGGCATCATT GAAACAGCAA

2581 GGCTCTTACA AGTTTTCACA AAAATCTATG AACTAGACAA AACAGTAACT GCAGAAAAAA

2641 GTCCTATATG TGCAAAAAAA TGCTTGATGA TTTGTAATGA GATTGAGGAG GTTTCGAGAC

2701 AGGCACCAAA GTTTTTACAA ATGGATATTT CCACCACCGC TCTAACCAAT TTGTTGAAGG

2761 AACACCCTTG GCTATCCTTT ACAAGATTCG AACTGAAGTG GAAACAGTTG TCTCTTATCA

2821 TTTATGTATT AAGAGATTTT TTCACTAATT TTACCCAGAA AAAGTCACAA CTAGAACAGG

2881 ATCAAAATGA TCATCAAAGT TATGAAGTTA AACGATGCTC CATCATGTTA AGCGATGCAG

2941 CACAAAGAAC TGTTATGTCT GTAAGTAGCT ATATGGACAA TCATAATGTC ACCCCATATT

3001 TTGCCTGGAA TTGTTCTTAT TACTTGTTCA ATGCAGTCCT AGTACCCATA AAGACTCTAC

3061 TCTCAAACTC AAAATCGAAT GCTGAGAATA ACGAGACCGC ACAATTATTA CAACAAATTA

3121 ACACTGTTCT GATGCTATTA AAAAAACTGG CCACTTTTAA AATCCAGACT TGTGAAAAAT

3181 ACATTCAAGT ACTGGAAGAG GTATGTGCGC CGTTTCTGTT ATCACAGTGT GCAATCCCAT

3241 TACCGCATAT CAGTTATAAC AATAGTAATG GTAGCGCCAT TAAAAATATT GTCGGTTCTG

3301 CAACTATCGC CCAATACCCT ACTCTTCCGG AGGAAAATGT CAACAATATC AGTGTTAAAT

3361 ATGTTTCTCC TGGCTCAGTA GGGCCTTCAC CTGTGCCATT GAAATCAGGA GCAAGTTTCA

3421 GTGATCTAGT CAAGCTGTTA TCTAACCGTC CACCCTCTCG TAACTCTCCA GTGACAATAC

3481 CAAGAAGCAC ACCTTCGCAT CGCTCAGTCA CGCCTTTTCT AGGGCAACAG CAACAGCTGC

3541 AATCATTAGT GCCACTGACC CCGTCTGCTT TGTTTGGTGG CGCCAATTTT AATCAAAGTG

3601 GGAATATTGC TGATAGCTCA TTGTCCTTCA CTTTCACTAA CAGTAGCAAC GGTCCGAACC
3661 TCATAACAAC TCAAACAAAT TCTCAAGCGC TTTCACAACC AATTGCCTCC TCTAACGTTC
3721 ATGATAACTT CATGAATAAT GAAATCACGG CTAGTAAAAT TGATGATGGT AATAATTCAA
3781 AACCACTGTC ACCTGGTTGG ACGGACCAAA CTGCGTATAA CGCGTTTGGA ATCACTACAG
3841 GGATGTTTAA TACCACTACA ATGGATGATG TATATAACTA TCTATTCGAT GATGAAGATA
3901 CCCCACCAAA CCCAAAAAAA GAGTAAAATG AATCGTAGAT ACTGAAAAAC CCCGCAAGTT
3961 CACTTCAACT GTGCATCGTG CACCATCTCA ATTTCTTTCA TTTATACATC GTTTTGCCTT
4021 CTTTTATGTA ACTATACTCC TCTAAGTTTC AATCTTGGCC ATGTAACCTC TGATCTATAG
4081 AATTTTTTAA ATGACTAGAA TTAATGCCCA TCTTTTTTTT GGACCTAAAT TCTTCATGAA
4141 AATATATTAC GAGGGCTTAT TCAGAAGCTT ATCGATACCG TCGACTAAAG CCAAATAGAA
4201 AATTATTCAG TTCCTGGCTT AAGTTTTTAA AAGTGATATT ATTTATTTGG TTGTAACCAA
4261 CCAAAAGAAT GTAAATAACT AATACATAAT TATGTTAGTT TTAAGTTAGC AACAAATTGA
4321 TTTTAGCTAT ATTAGCTACT TGGTTAATAA ATAGAATATA TTTATTTAAA GATAATTGCG
4381 TTTTTATTGT CAGGGAGTGA GTTTGCTTAA AAACTCGTTT AGATCCACTA GTTCTAGATA
4441 ACTTCGTATA GCATACATTA TACGAAGTTA TAGCTTCTGA TGGAATTAGA ACTTGGCAAA
4501 ACAATACTGA GAATGAAGTG TATGTGGAAC AGAGGCTGCT GATCTCGTTC TTCAGGCTAT
4561 GAAACTGACA CATTTGGAAA CCACAGTACT TAGAACCACA AAGTGGGAAT CAAGAGAAAA
4621 ACAATGATCC CACGAGAGAT CGAATTCTAG TATGTATGTA AGTTAATAAA ACCCATTTTT
4681 GCGGAAAGTA GATAAAAAAA ACATTTTTTT TTTTTACTGC ACTGGATATC ATTGAACTTA
4741 TCTGATCAGT TTTAAATTTA CTTCGATCCA AGGGTATTTG ATGTACCAGG TTCTTTCGAT
4801 TACCTCTCAC TCAAAATGAC ATTCCACTCA AAGTCAGCGC TGTTTGCCTC CTTCTCTGTC
4861 CACAGAAATA TCGCCGTCTC TTTCGCCGCT GCGTCCGCTA TCTCTTTCGC CACCGTTTGT
4921 AGCGTTACGT AGCGTCAATG TCCGCCTTCA GTTGCATTTT GTCAGCGGTT TCGTGACGAA
4981 GCTCCAAGCG GTTTACGCCA TCAATTAAAC ACAAAGTGCT GTGCCAAAAC TCCTCTCGCT
5041 TCTTATTTTT GTTTGTTTTT TGAGTGATTG GGGTGGTGAT TGGTTTTGGG TGGGTAAGCA
5101 GGGGAAAGTG TGAAAAATCC CGGCAATGGG CCAAGAGGAT CAGGAGCTAT TAATTCGCGG
5161 AGGCAGCAAA CACCCATCTG CCGAGCATCT GAACAATGTG AGTAGTACAT GTGCATACAT
5221 CTTAAGTTCA CTTGATCTAT AGGAACTGCG ATTGCAACAT CAAATTGTCT GCGGCGTGAG
5281 AACTGCGACC CACAAAAATC CCAAACCGCA ATTGCACAAA CAAATAGTGA CACGAAACAG
5341 ATTATTCTGG TAGCTGTTCT CGCTATATAA GACAATTTTT GAGATCATAT CATGATCAAG

5401 ACATCTAAAG GCATTCATTT TCGACTATAT TCTTTTTTAC AAAAAATATA ACAACCAGAT
5461 ATTTTAAGCT GATCCTAGAT GCACAAAAAA TAAATAAAAG TATAAACCTA CTTCGTAGGA
5521 TACTTCGGGG TACTTTTTGT TCGGGGTTAG ATGAGCATAA CGCTTGTAGT TGATATTTGA
5581 GATCCCCTAT CATTGCAGGG TGACAGCGGA GCGGCTTCGC AGAGCTGCAT TAACCAGGGC
5641 TTCGGGCAGG CCAAAAACTA CGGCACGCTC CGGCCACCCA GTCCGCCGGA GGACTCCGGT
5701 TCAGGGAGCG GCCAACTAGC CGAGAACCTC ACCTATGCCT GGCACAATAT GGACATCTTT
5761 GGGGCGGTCA ATCAGCCGGG CTCCGGATGG CGGCAGCTGG TCAACCGGAC ACGCGGACTA
5821 TTCTGCAACG AGCGACACAT ACCGGCGCCC AGGAAACATT TGCTCAAGAA CGGTGAGTTT
5881 CTATTCGCAG TCGGCTGATC TGTGTGAAAT CTTAATAAAG GGTCCAATTA CCAATTTGAA
5941 ACTCAGTTTG CGGCGTGGCC TATCCGGGCG AACTTTTGGC CGTGATGGGC AGTTCCGGTG
6001 CCGGAAAGAC GACCCTGCTG AATGCCCTTG CCTTTCGATC GCCGCAGGGC ATCCAAGTAT
6061 CGCCATCCGG GATGCGACTG CTCAATGGCC AACCTGTGGA CGCCAAGGAG ATGCAGGCCA
6121 GGTGCGCCTA TGTCCAGCAG GATGACCTCT TTATCGGCTC CCTAACGGCC AGGGAACACC
6181 TGATTTTCCA AGCCATGGTG CGGATGCCAC GACATCTGAC CTATCGGCAG CGAGTGGCCC
6241 GCGTGGATCA GGTGATCCAG GAGCTTTCGC TCAGCAAATG TCAGCACACG ATCATCGGTG
6301 TGCCCGGCAG GGTGAAAGGT CTGTCCGGCG GAGAAAGGAA GCGTCTGGCA TTCGCCTCCG
6361 AGGCTCTAAC CGATCCGCCG CTTCTGATCT GCGATGAGCC CACCTCCGGA CTGGACTCCT
6421 TTACCGCCCA CAGCGTCGTC CAGGTGCTGA AGAAGCTGTC GCAGAAGGGC AAGACCGTCA
6481 TCCTGACCAT TCATCAGCCG TCTTCCGAGC TGTTTGAGCT CTTTGACAAG ATCCTTCTGA
6541 TGGCCGAGGG CAGGGTAGCT TTCTTGGGCA CTCCCAGCGA AGCCGTCGAC TTCTTTTCCT
6601 AGTGAGTTCG ATGTGTTTAT TAAGGGTATC TAGTATTACA TAACATCTCA ACTCCTATCC
6661 AGCGTGGGTG CCCAGTGTCC TACCAACTAC AATCCGGCGG ACTTTTACGT ACAGGTGTTG
6721 GCCGTTGTGC CCGGACGGGA GATCGAGTCC CGTGATCGGA TCGCCAAGAT ATGCGACAAT
6781 TTTGCCATTA GCAAAGTAGC CCGGGATATG GAGCAGTTGT TGGCCACCAA AAATCTGGAG
6841 AAGCCACTGG AGCAGCCGGA GAATGGGTAC ACCTACAAGG CCACCTGGTT CATGCAGTTC
6901 CGGGCGGTCC TGTGGCGATC CTGGCTGTCG GTGCTCAAGG AACCACTCCT CGTAAAAGTG
6961 CGACTTATTC AGACAACGGT GAGTGGTTCC AGTGGAAACA AATGATATAA CGCTTACAAT
7021 TCTTGGAAAC AAATTCGCTA GATTTTAGAT AGAATTGCCT GATTCCACAC CCTTCTTAGT
7081 TTTTTTCAAT GAGATGTATA GTTTATAGTT TTGCAGAAGA TAAATAAATT TCATTTAACT
7141 CGCGAATATT AATGAGATGC GAGTAACATT TTAATTTGCA GATGGTTGCC ATCTTGATTG

7201 GCCTCATCTT TTTGGGCCAA CAACTCACGC AAGTGGGTGT GATGAATATC AACGGAGCCA
7261 TCTTCCTCTT CCTGACCAAC ATGACCTTTC AAAACGTCTT TGCCACGATA AATGTAAGTC
7321 ATGTTTAGAA TACATTTGCA TTTCAATAAT TTACTAACTT TCTAATGAAT CGATTCGATT
7381 TAGGTGTTCA CCTCAGAGCT GCCAGTTTTT ATGAGGGAGG CCCGAAGTCG ACTTTATCGC
7441 TGTGACACAT ACTTTCTGGG CAAAACGATT GCCGAATTGC CGCTTTTTCT CACAGTGCCA
7501 CTGGTCTTCA CGGCGATTGC CTATCCGATG ATCGGACTGC GGGCCGGAGT GCTGCACTTC
7561 TTCAACTGCC TGGCGCTGGT CACTCTGGTG GCCAATGTGT CAACGTCCTT CGGATATCTA
7621 ATATCCTGCG CCAGCTCCTC GACCTCGATG GCGCTGTCTG TGGGTCCGCC GGTTATCATA
7681 CCATTCCTGC TCTTTGGCGG CTTCTTCTTG AACTCGGGCT CGGTGCCAGT ATACCTCAAA
7741 TGGTTGTCGT ACCTCTCATG GTTCCGTTAC GCCAACGAGG GTCTGCTGAT TAACCAATGG
7801 GCGGACGTGG AGCCGGGCGA AATTAGCTGC ACATCGTCGA ACACCACGTG CCCCAGTTCG
7861 GGCAAGGTCA TCCTGGAGAC GCTTAACTTC TCCGCCGCCG ATCTGCCGCT GGACTACGTG
7921 GGTCTGGCCA TTCTCATCGT GAGCTTCCGG GTGCTCGCAT ATCTGGCTCT AAGACTTCGG
7981 GCCCGACGCA AGGAGTAGCC GACATATATC CGAAATAACT GCTTGTTTTT TTTTTTTACC
8041 ATTATTACCA TCGTGTTTAC TGTTTATTGC CCCCTCAAAA AGCTAATGTA ATTATATTTG
8101 TGCCAATAAA AACAAGATAT GACCTATAGA ATACAAGTAT TTCCCCTTCG AACATCCCCA
8161 CAAGTAGACT TTGGATTTGT CTTCTAACCA AAAGACTTAC ACACCTGCAT ACCTTACATC
8221 AAAAACTCGT TTATCGCTAC ATAAAACACC GGGATATATT TTTTATATAC ATACTTTTCA
8281 AATCGCGCGC CCTCTTCATA ATTCACCTCC ACCACACCAC GTTTCGTAGT TGCTCTTTCG
8341 CTGTCTCCCA CCCGCTCTCC GCAACACATT CACCTTTTGT TCGACGACCT TGGAGCGACT
8401 GTCGTTAGTT CCGCGCGATT CGGTTCGCTC AAATGGTTCC GAGTGGTTCA TTTCGTCTCA
8461 ATAGAAATTA GTAATAAATA TTTGTATGTA CAATTTATTT GCTCCAATAT ATTTGTATAT
8521 ATTTCCCTCA CAGCTATATT TATTCTAATT TAATATTATG ACTTTTTAAG GTAATTTTTT
8581 GTGACCTGTT CGGAGTGATT AGCGTTACAA TTTGAACTGA AAGTGACATC CAGTGTTTGT
8641 TCCTTGTGTA GATGCATCTC AAAAAAATGG TGGGCATAAT AGTGTTGTTT ATATATATCA
8701 AAAATAACAA CTATAATAAT AAGAATACAT TTAATTTAGA AAATGCTTGG ATTTCACTGG
8761 AACTAGAATT CATAACTTCG TATAGCATAC ATTATACGAA GTTATGGATC CTAGTTCTCT
8821 CTCTCTCTCT CTTATCTATC GCTACTTGGT TGGCGCGCTC TCGCGCTCTC TTTGTGTGCG
8881 TGTGGGCAGT GTGTTTTTTG TTGTTTTGCG CTTTATGTGT TGTATTTTGT GTGTTTGGCC
8941 GAAGTATTTA AAACAAAAGT GCAGCGGAAA TAGTTAATAA CAAAATATTA GTCGACGGTA

9001 GCGGTCCGGT TGTTTTCGTG CTCATCGCGA GTACGTATTT ATTTTTAAAA AATAATAAAC

9061 GCGGCAAACA CAATCACACA AATGTGCAGG CACAGTGGGA CAAAGTGCGC TAAATTTGGA

9121 TATGTAATAA ATCCAAAACC AAAAAGAAAA CAAAAAACGG TAAAATATTT TACTGTTGTT

9181 AAAATTCGAT CATTCATTAT TCGCTGCATG AATTAGCTTT GCGTACTCGC AAATTATTAA

9241 AAATAAAACT TTAAAAATAA TTTCGTCTAA TTAATATTAT GAGTTAATTC AAACCCCACG

9301 GACATGCTAA GGGTTAATCA ACAATCATAT CGCTGTCTCA CTCAGACTCA ATACGACACT

9361 CAGAATACTA TTCCTTTCAC TCGCACTTAT TGCAAGCATA CGTTAAGTGG ATGTCTCTTG

9421 CCGACGGGAC CACCTTATGT TATTTCATCA TG

RED: END OF TIR

GREEN: SP1 SEQUENCING PRIMER ANNEALING SITE

DARK BLUE: PLAC1 ANNEALING SITE

TEAL: PLAC4 ANNEALING SITE

BLUE: HPAII SITE

BLACK: SAU3AI SITE

OCHRE: HSP70 PROMOTOR, WILL ALIGN TO THE 4 HSP70 GENES ON 3R

YELLOW: ANNEALS TO KIRRE (THIS IS 3’ OF WHITE, IN THE 3’ OF THE P)

GREY: ANNEALS TO WHITE

BROWN: ANNA ANNEALING SITE

DARK GREEN: BERTA ANNEALING SITE
