# Supplementary figures and images for: Generation of LexA enhancer-trap lines in Drosophila by an international scholastic network
Source: G3 (Bethesda). 2023 Jun 6;13(9):jkad124. doi: 10.1093/g3journal/jkad124 (PMC10468311; doi:10.1093/g3journal/jkad124)

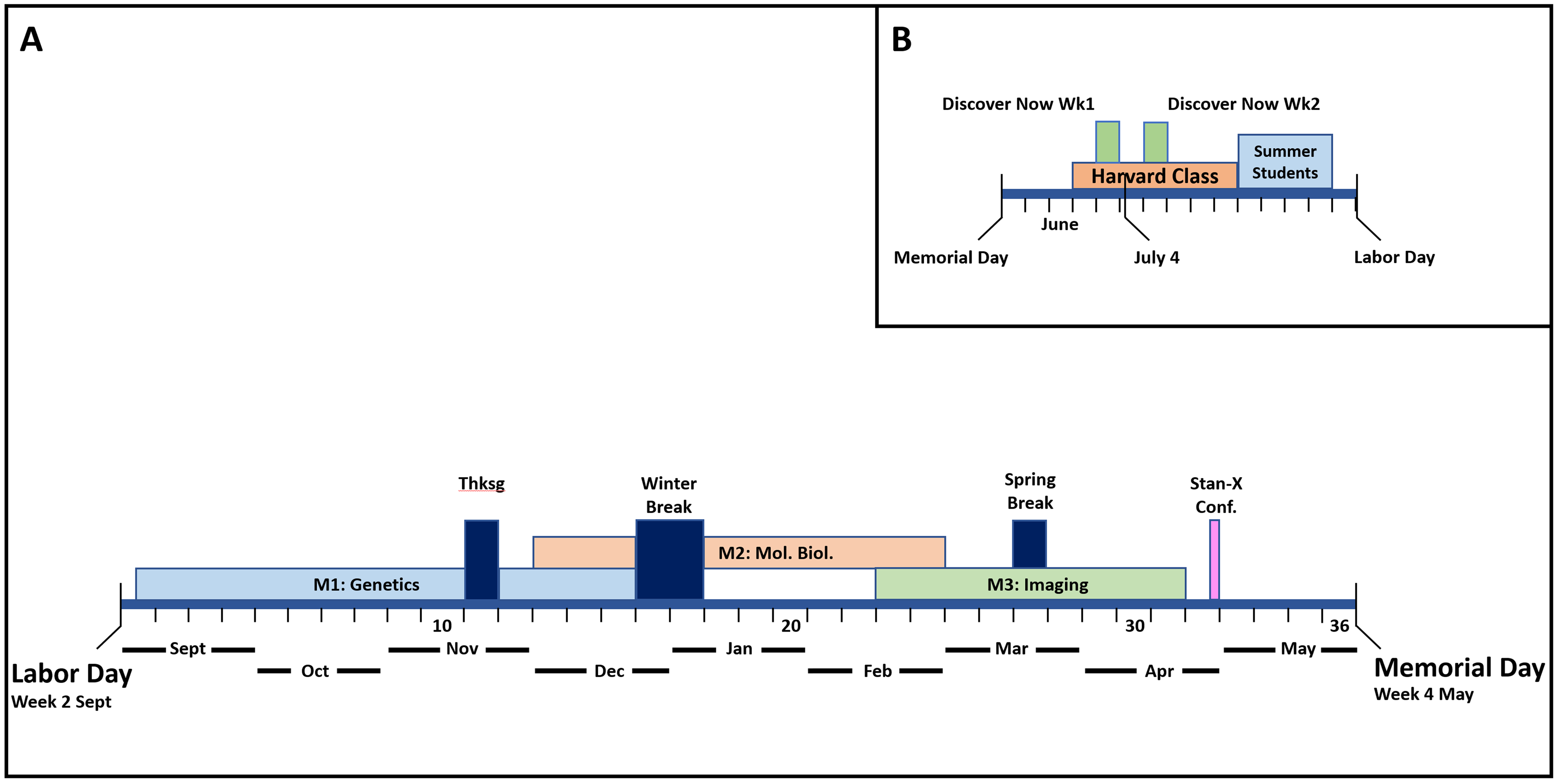

Supplement: jkad124_Supplementary_Data [file jkad124_supplementary_data.zip › Figure_S1_G3-2023-404154.tif]

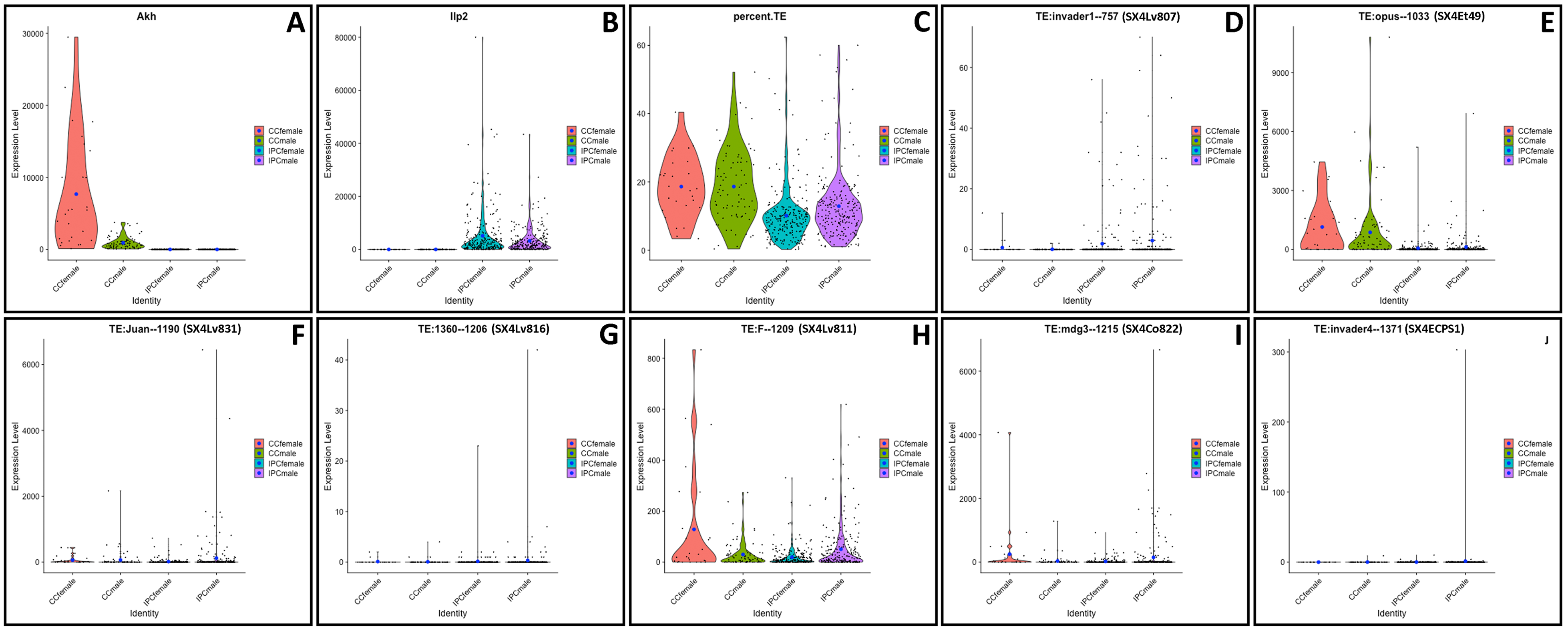

Supplement: jkad124_Supplementary_Data [file jkad124_supplementary_data.zip › Figure_S2_G3-2023-404154.tif]
